# Investigating the role of CpG islands and DNA methylation at 3′UTRs in cancer

**DOI:** 10.26508/lsa.202503234

**Published:** 2026-07-24

**Authors:** Claire Wilson, Aditi Kanhere

**Affiliations:** Department of Molecular and Clinical Cancer Medicine, Institute of Systems, Molecular and Integrative Biology, University of Liverpool , Liverpool, UK

## Abstract

DNA methylation is one of the most important epigenetic processes that regulates gene expression. Although the human genome is predominantly methylated, CpG-rich regions known as CpG islands (CGIs) are typically unmethylated and are often sites of transcriptional control. Although the importance of CGIs and DNA methylation at the promoters is well understood, the significance of CGIs at the 3′ ends remains largely unexplored. In this study, we characterised CGIs overlapping 3′ untranslated regions (3′UTRs), compared them with CGIs located at the promoters, and examined their association with gene expression in cancer. We found that 3% of CGIs overlap 3′UTRs and have no annotated promoters in the vicinity. Unlike promoter CGIs, 3′UTR CGIs are shorter, less CpG-dense, but evolutionarily conserved. Although promoter CGIs typically stay unmethylated, those in 3′UTRs are often methylated and enriched for transcription factors like ZFP57, which target methylated motifs. These regions are linked to cancer signalling pathways, and their differential methylation in tumours correlates with gene expression changes, suggesting an underexplored regulatory role in cancer.

## Abstract (precis)

This study characterises CpG islands at 3′UTRs as highly conserved, frequently methylated, and linked to cancer-related gene regulation.

## Introduction

DNA methylation is a major epigenetic process that regulates gene expression, tissue differentiation, and development (Moore et al, 2013). The majority of DNA methylation in the human genome occurs at CpG dinucleotides, where cytosine is followed by a guanine nucleotide (Moore et al, 2013). However, regions with a high density of CpG dinucleotides and elevated GC content, known as CpG islands (CGIs), are typically protected from DNA methylation (Bird, 1986). CGIs are frequently found at promoter regions (Moore et al, 2013; Steinhaus et al, 2020), with roughly 70% of gene promoters exhibiting high CpG content (Saxonov et al, 2006).

CGIs at gene promoters play a key role in regulating gene expression. Promoter CGIs are thought to be actively protected from methylation, promote open chromatin structure and increase the accessibility of DNA (Long et al, 2016). This facilitates the binding of transcription factor (TF) and RNA polymerase to promoters, leading to increased gene expression. Unmethylated promoter CGIs are also often associated with H3K4me3, a histone modification mark linked to transcription initiation (Hughes et al, 2020). H3K4me3 is proposed to inhibit methylation at promoter CGIs by preventing the binding and activity of DNA methyltransferases responsible for establishing DNA methylation (Hughes et al, 2020). On the other hand, methylation of promoter CGIs impairs TF binding and promotes the recruitment of repressive methyl-binding proteins, leading to stable gene silencing (Nishiyama & Nakanishi, 2021). In cancer, DNA methylation has been identified as a key epigenetic mechanism that influences gene expression changes favouring cell proliferation. Cancer cells not only often exhibit a global loss of CpG methylation but also show locus-specific hypermethylation at promoter CGIs, often leading to silencing of tumour suppressor genes and promoting uncontrolled cell proliferation (Nishiyama & Nakanishi, 2021).

Although most of the research on CGIs has focused on promoter CGIs, a significant number of CGIs are also located within the gene body and intergenic regions (Cain et al, 2022). Unlike promoter CGIs, intragenic CGIs often become methylated during development and differentiation (Jeziorska et al, 2017). Methylation of intragenic CGIs occurs in a tissue-specific manner, suggesting the process is important for gene regulation (Jeziorska et al, 2017). Numerous mechanisms by which intragenic CGIs can influence gene expression have been proposed. For example, intragenic CGIs have been shown to act as alternative promoters, with methylation of these CGIs impacting the expression of the host genes, antisense RNAs, or microRNAs (Cain et al, 2022). In addition, intragenic CGIs have been implicated in pre-mRNA processing mechanisms including alternative polyadenylation (polyA) (Amante et al, 2020) and alternative splicing (Maunakea et al, 2013).

The 3′ untranslated region (3′UTR) is a non-coding segment located at the 3′ end of mRNA, immediately downstream of the coding region (Mayr, 2019). 3′UTRs contain *cis*-regulatory sequences that can be targeted by *trans*-acting factors such as microRNA machinery and RNA-binding proteins (Mayya & Duchaine, 2019). These features of 3′UTRs help to regulate gene expression through post-transcriptional mechanisms, including controlling mRNA stability, translation, and localisation (Mayr, 2019). Variations in 3′UTR sequence and length can therefore impact gene expression. In the human genome, there is a high diversity of 3′UTRs, with over 60% of human genes displaying alternative 3′UTR isoforms (Derti et al, 2012; Hoque et al, 2013). This 3′UTR diversity contributes to the transcriptome landscape, and dysregulation of 3′UTR variants can drive the development and progression of diseases such as cancer (Chan et al, 2023; Hong & Jeong, 2023).

A few prior studies investigating CGI distribution in the genome have identified CGIs within 3′UTRs, although these studies were not focused predominantly on the 3′UTRs and did not analyse methylation patterns at these CGIs (Medvedeva et al, 2010; Chen et al, 2021; Sommerer et al, 2022). One study investigating a single 3′UTR CGI found methylation at this site can regulate alternative polyA and cleavage (Nanavaty et al, 2020). This study showed that demethylation of the 3′UTR CGI enables TF CTCF to bind the unmethylated site. In turn, this binding facilitates recruitment of the cohesin complex, leading to chromatin loop formation that blocks transcriptional elongation and promotes the use of proximal polyA sites (Nanavaty et al, 2020). In addition, a study looking at the 3′UTR of the *HAVCR2* gene found that 3′UTR methylation might be associated with the up-regulation of this gene in cancer cell lines (McGuire et al, 2019).

Therefore, it appears that CGIs located within the 3′UTR and their methylation may also play an important role in regulating gene expression. In this study, we aimed to characterise CGIs located within 3′UTRs and examine their association with gene regulation in cancer.

## Results

### CGIs overlapping 3′UTR exhibit distinct features compared with promoter CGIs

**Figure 1.**
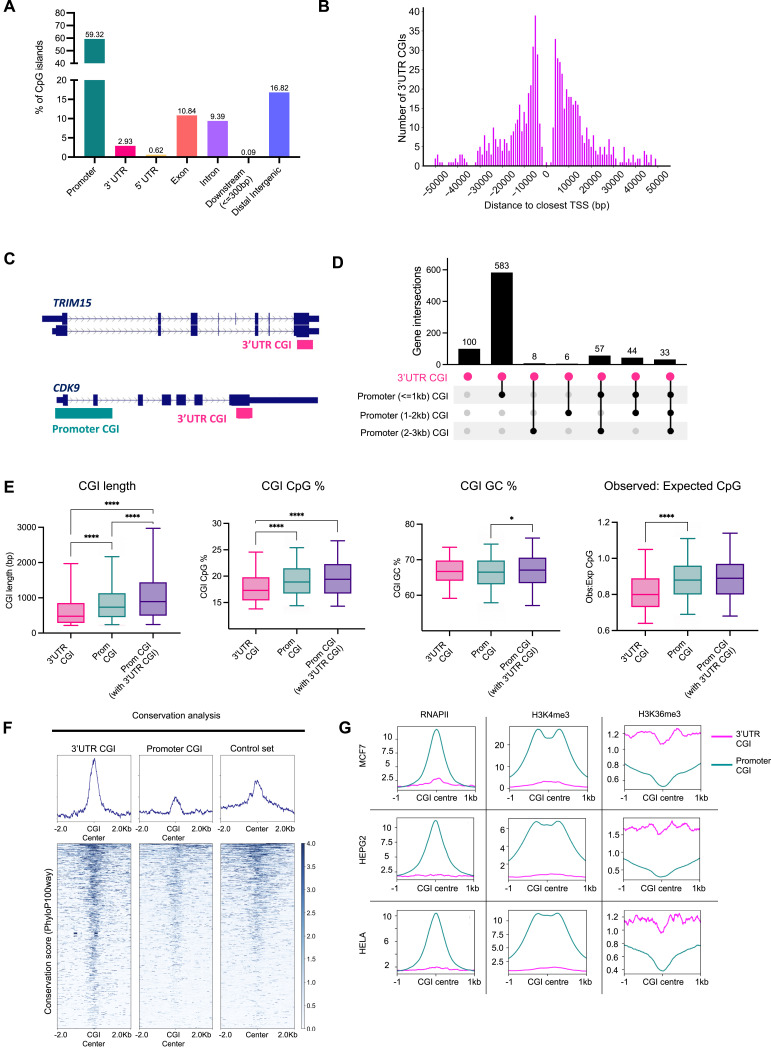
Characterisation of 3′UTR CGIs. **(A)** Bar chart showing the genomic distribution of CGIs. The percentage of CGIs associated with each genomic annotation is shown at the top of bars. **(B)** Histogram of the distance of 3′UTR CGIs from known TSSs. **(C)** Examples of genes containing 3′UTR CGIs with or without promoter CGI. **(D)** UpSet plot showing the number of genes with 3′UTR CGIs that also contain promoter CGIs. The distance in brackets (<=1, 1, 2kb) indicates the distance of the CGI from the nearest TSS. **(E)** Box-and-whisker plot (5–95% confidence interval) showing distribution of the CGI length, the percentage of CpGs within the CGI, the percentage of GC within the CGI, and the observed-to-expected CpG ratio within 3′UTR CGIs, promoter CGIs (Prom CGI), and promoter CGIs associated with a gene also containing 3′UTR CGIs (“Prom CGI [with 3′UTR CGI]”). *P < 0.05; ****P < 0.0001 (Brown–Forsythe and Welch ANOVA test). **(F)** Metagene plot and heatmaps showing the conservation (PhyloP100way) score of 3′UTR CGIs, promoter CGIs (subset of equal number to 3′UTR CGIs), and random control sites with similar lengths and overlaps with genomic annotations as 3′UTR CGIs. **(G)** Metagene plots showing the enrichment of RNAPII, H3K4me3, and H3K36me3 centred on CGIs in 3′UTRs and promoters in MCF7 (breast cancer cell line), HEPG2 (hepatocellular carcinoma cell line), and HeLa (cervical cancer cell line).

We first sought to characterise CGIs overlapping 3′UTRs and compare them with promoter CGIs. We identified 909 CGIs (∼3% of all CGIs) that overlap 3′UTRs of genes (3′UTR CGIs) and do not overlap or are in the vicinity of any promoters (Fig 1A and B). Although these CGIs are classified based on their overlap with annotated 3′UTRs, they spanned to a varied extent into neighbouring genic features, such as terminal coding exons or introns, as well as intergenic regions.

We first assessed whether genes harbouring 3′UTR-associated CGIs also contain CGIs at their promoters. Based on their association with promoter CGIs, the genes with 3′UTR CGIs can be divided into two main categories as shown in Fig 1C: (1) those with CGIs overlapping 3′UTR and no CGIs in the promoter (e.g., *TRIM15* gene), and (2) those with CGIs overlapping both the promoter and the 3′UTR (e.g., *CDK9*). Only a small subset of genes containing 3′UTR CGIs (100 genes) display no CGIs at the promoter. A vast majority of genes (731 genes) with 3′UTR CGIs also contain promoter CGIs (Fig 1D).

**Figure S1.**
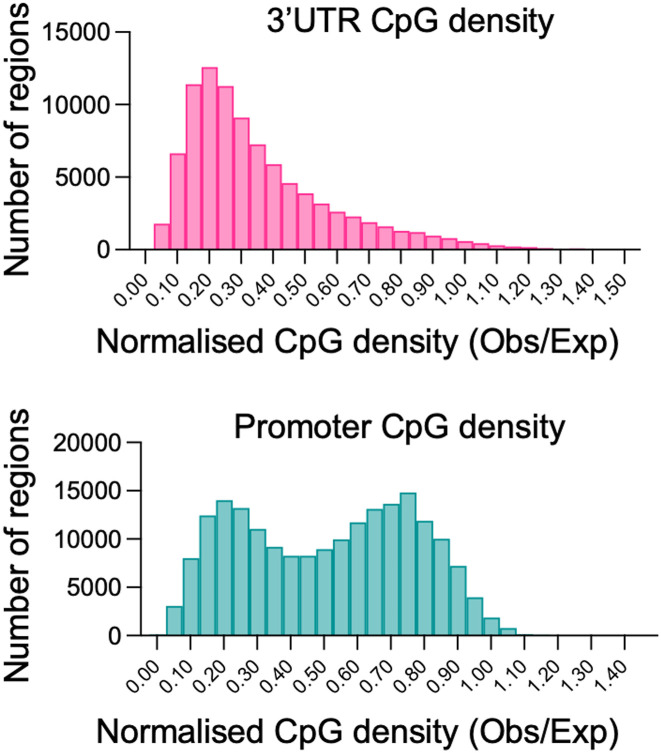
Comparison of CpG density between promoters and 3′UTRs. Histogram of normalised CpG densities across 3′UTRs and promoter regions. CpG density was calculated as the observed-to-expected ratio (OE = [number of CpGs]/[number of Cs × number of Gs] × length of the region in nucleotides).

To determine how 3′UTR CGIs differ from promoter CGIs, we compared their properties such as length, CpG density, and GC content (Fig 1E). This revealed that 3′UTR CGIs are significantly shorter and contain fewer CpG sites, suggesting they are structurally weaker than promoter CGIs. Given that most genes with 3′UTR CGIs also carry CGIs in their promoters, we next examined whether their promoter CGIs display distinctive properties compared with promoters of genes lacking 3′UTR CGIs. In genes with CGIs at both promoter and 3′UTRs, promoter CGIs were significantly longer, although their CpG density and GC content were comparable to other promoter CGIs (Fig 1E). Furthermore, a comparison of CpG density distributions across 3′UTRs revealed a unimodal pattern with a long right tail, in contrast to the bimodal distribution observed in promoters (Fig S1). This suggests that 3′UTR CGIs represent the high-density end of a continuous distribution rather than a distinct subclass as in case of promoters (Fig S1). We considered the possibility that the shorter length and reduced CpG density of 3′UTR CGIs might indicate these regions are associated with non-functional transcripts such as pseudogenes. To verify gene classifications, we obtained *gene_biotype* annotations from BioMart and validated them against GENCODE annotation data, confirming that genes harbouring 3′UTR CGIs in our dataset are exclusively protein-coding (Table S1).

Table S1. List of genes with 3′UTR CGIs, 3′UTR CGI coordinates, and promoter CGI coordinates.

**Figure S2.**
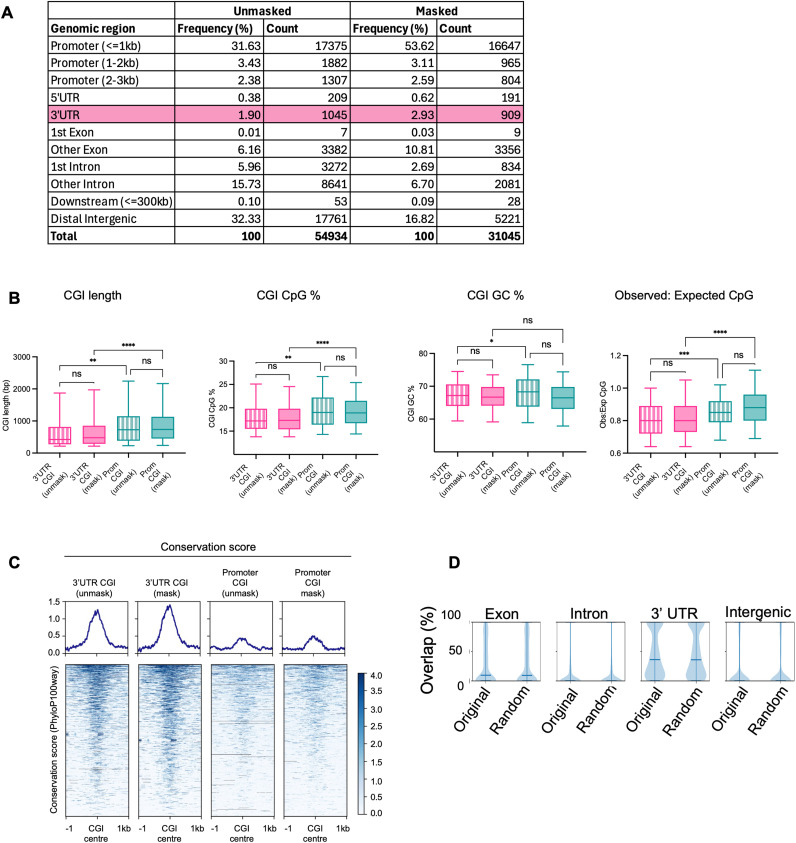
Characteristics of CGIs from UCSC unmasked CpG track. **(A)** Table showing the genomic distribution of CGIs in UCSC masked and unmasked CpG tracks. 3′UTR CGIs are highlighted in pink. **(B)** Box-and-whisker plot with 5–95% confidence interval showing the CGI length, the percentage of CpG within the CGI, the percentage of GC within the CGI, and the observed-to-expected CpG ratio within 3′UTR CGIs and promoter CGIs from UCSC masked and unmasked CpG tracks. *P < 0.05; **P < 0.01; ***P < 0.001; ****P < 0.0001 (Brown–Forsythe and Welch ANOVA test). **(C)** Metagene plot and heatmaps showing the conservation (PhyloP100way) score of 3′UTR CGIs and promoter CGIs (subset of equal number to 3′UTR CGIs) from UCSC masked and unmasked CpG tracks. **(D)** Box plots demonstrating that the control set effectively mirrors the genomic annotation overlap (exons and introns) observed in 3′ UTR CGIs.

We further assessed the potential impact of repeat masking on CGI detection (Fig S2A). CGI prediction using unmasked genome identified a small additional number of CGIs (136) in 3′UTRs corresponding to 220 genes of which nine were pseudogenes and predicted transcripts. A comparison of CGI properties between masked and unmasked genomes shows that the analysis is minimally influenced by repetitive elements (Fig S2A–C).

Given that 3′UTR CGIs are shorter and contain fewer CpG sites than promoter CGIs, we investigated their levels of evolutionary constraint compared with promoter CGIs. To test this, we examined their evolutionary conservation using PhyloP100way scores (calculated across 100 vertebrate species), centred on 3′UTR CGIs, promoter CGIs, and a control set (Fig 1F). Given that some CGIs also partially overlapped terminal coding exons, introns, and intergenic regions, in addition to 3′UTR, the control set was size-matched and also ensured a similar distribution of overlap with neighbouring features (Fig S2D, Materials and Methods). Remarkably, our analysis revealed that 3′UTR CGIs exhibit a high degree of sequence conservation similar to promoter CGIs (Fig 1F). Previous studies have shown that highly conserved regulatory elements involved in transcriptional termination and post-transcriptional regulation are present in 3′UTRs (Siepel et al, 2005). Therefore, the high conservation score of 3′UTR CGIs might merely reflect regulatory elements present in 3′UTRs rather than CGIs themselves. To rule out this possibility, we also compared the conservation of 3′UTR CGIs with that of the matched control set (Materials and Methods, Fig S2D). The 3′UTR CGIs remained more conserved than the control set (Fig 1F).

**Figure S3.**
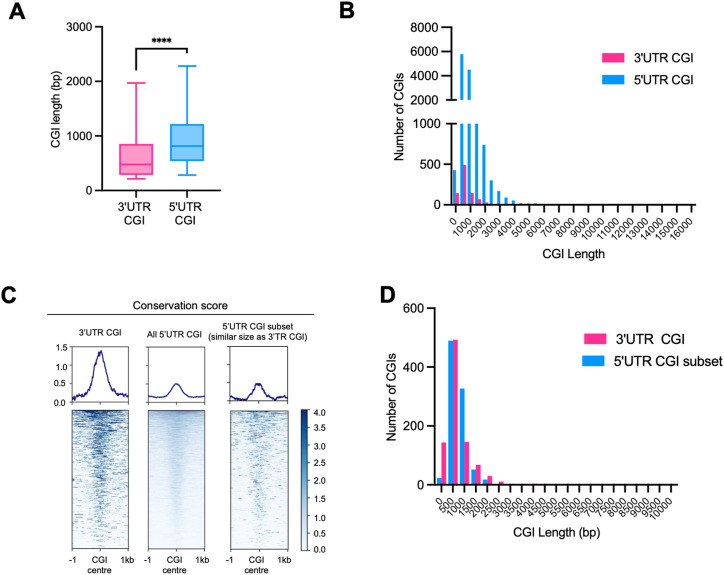
Conservation comparison between 3′UTR CGIs and 5′UTR CGIs. **(A)** Box-and-whisker plot with 5–95% confidence interval showing the CGI length of 3′UTRs and 5′UTR CGIs. ****P < 0.0001 (*t* test). **(B)** Histogram showing the distribution of CGI length across 3′UTR and 5′UTR CGIs. **(C)** Metagene plot and heatmaps showing the conservation (PhyloP100way) score of 3′UTR CGIs, 5′UTR CGIs, and a subset of 5′UTR CGIs, which are a similar size in length to 3′UTR CGIs. **(D)** Histogram showing the distribution of 3′UTRs and a selected subset of 5′UTR CGIs matched in length to 3′UTR CGIs.

To determine whether the unique characteristics of 3′UTR CGIs were a general feature of UTR-localised elements, we performed a targeted comparison with CGIs overlapping 5′UTRs. In our primary genome-wide characterisation, most of the 5′UTR-associated CGIs were assigned to the “Promoter” category because of our hierarchical priority (Promoter > 3′UTR > 5′UTR). This hierarchy was established to ensure that the identified 3′UTR CGIs did not overlap or lie in the immediate vicinity of promoters. However, to address whether the high conservation observed at the 3′ end was simply a consequence of a UTR-centric genomic environment, we specifically extracted of CGIs with 5′UTR overlap for a focused subanalysis. This comparison revealed that consistent with our earlier results, 3′UTR CGIs remain significantly shorter than 5′UTR CGIs (Fig S3A and B). Crucially, even after selecting 5′UTR CGIs that matched in length distribution to that of the 3′UTR CGIs, the 3′UTR elements exhibited higher levels of evolutionary conservation across all groups (Fig S3C and D). These data indicate that the characteristics of 3′UTR CGIs are not a general attribute of all UTR-associated CGIs.

**Figure S4.**
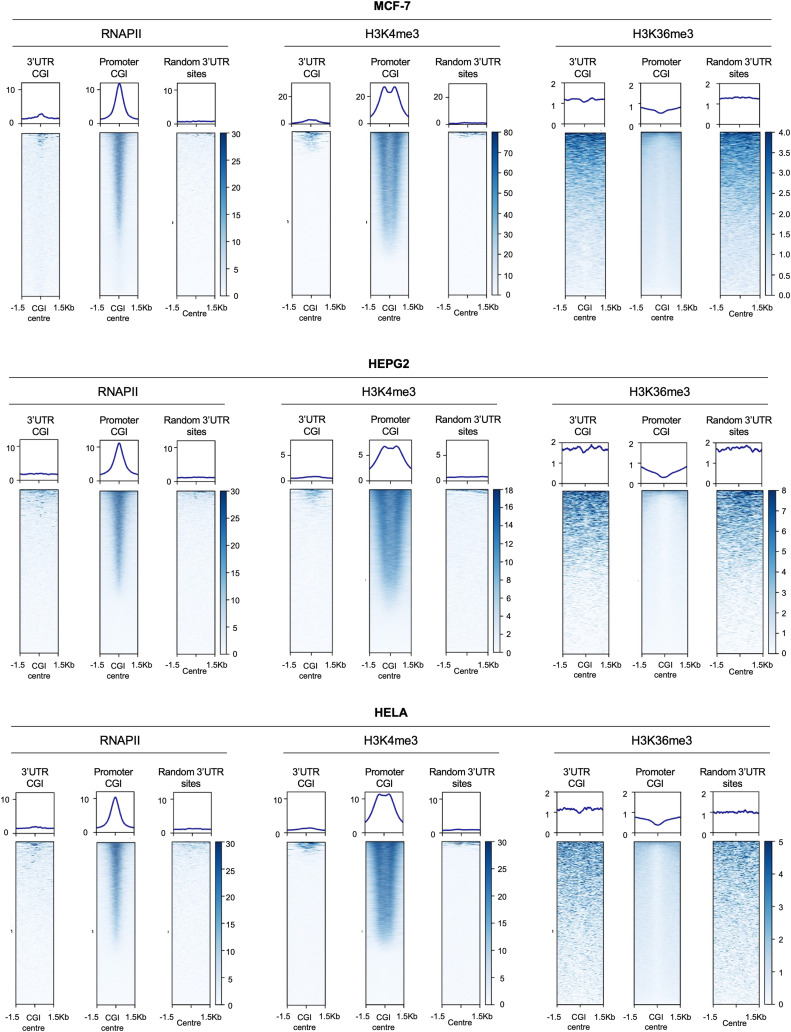
RNAPII, H3K4me3, and H3K36me3 enrichment at 3′UTR and promoter CGIs, and random 3′UTRs. Metagene plots and heatmaps showing the enrichment of RNAPII, H3K4me3, and H3K36me3 centred on CGIs in 3′UTRs and promoters, as well as randomly selected regions from 3′UTRs in MCF7 (breast cancer cell line), HEPG2 (hepatocellular carcinoma cell line), and HeLa (cervical cancer cell line) cells.

**Figure S5.**
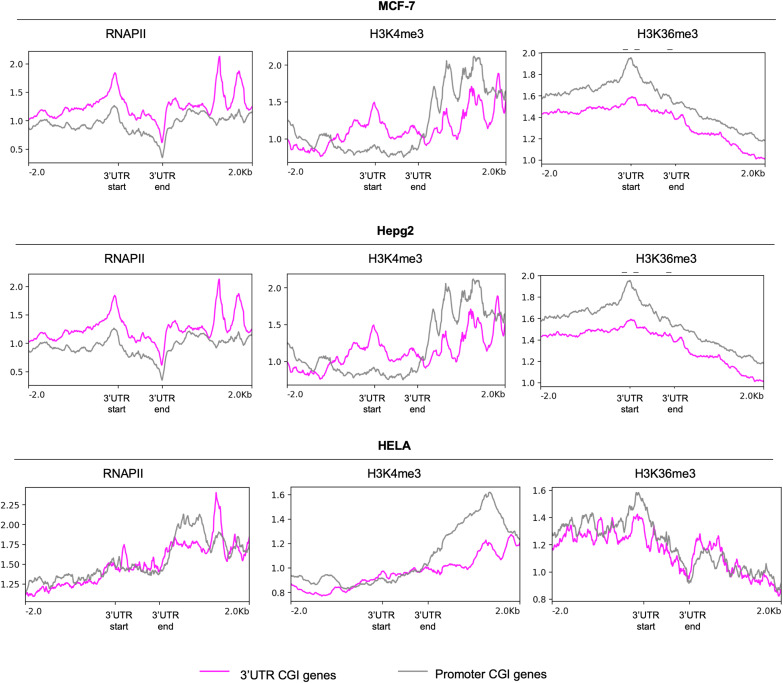
RNAPII, H3K4me3, and H3K36me3 enrichment across 3′UTRs. Metagene plots showing the enrichment of RNAPII, H3K4me3, and H3K36me3 across 3′UTRs in genes containing 3′UTR CGIs and in genes containing only promoter CGIs (subset matched in number to the 3′UTR CGI group) in MCF7 (breast cancer cell line), HEPG2 (hepatocellular carcinoma cell line), and HeLa (cervical cancer cell line).

We next examined whether 3′UTR CGIs, like promoter CGIs, might also function as transcription start sites (TSSs) and initiate expression of a nearby transcript. To explore the potential role of 3′UTR CGIs as sites of transcription initiation, we analysed RNA polymerase II (RNAPII) binding and the enrichment of the histone modification H3K4me3 across several cell lines (Figs 1G, S4, and S5). The analysis was performed by plotting ChIP-seq enrichment both centred on the CGIs (Figs 1G and S4) and across the entire CGI regions (Fig S5), as well as for non-CGI 3′UTRs as a control. 3′UTR CGIs showed little or no enrichment for RNAPII and H3K4me3, in contrast to promoter CGIs, which displayed strong enrichment for both. We also looked at the histone mark H3K36me3, which is associated with transcription elongation and is correlated with DNA methyltransferase 3B–mediated methylation (Hahn et al, 2011). We observed low levels of H3K36me3 in promoter CGIs, which correlates with these sites being predominantly sites of transcription initiation rather than transcription elongation. At 3′UTR CGI sites, H3K36me3 showed higher enrichment than at the promoter CGIs, but it is comparable to a control set of non-CGI 3′UTRs. This pattern aligns with a previous report (Akhtar et al, 2013) showing that H3K36me3 is enriched at conserved 3′ gene ends, reflecting its association with transcriptional elongation and RNA processing. Together, the data show 3′UTR CGIs differ from promoter CGIs and lack the histone modification signatures typically associated with the alternative TSS or sites of enhanced elongation.

### Genes with 3′UTR CGIs are associated with cancer-related signalling pathways

**Figure 2.**
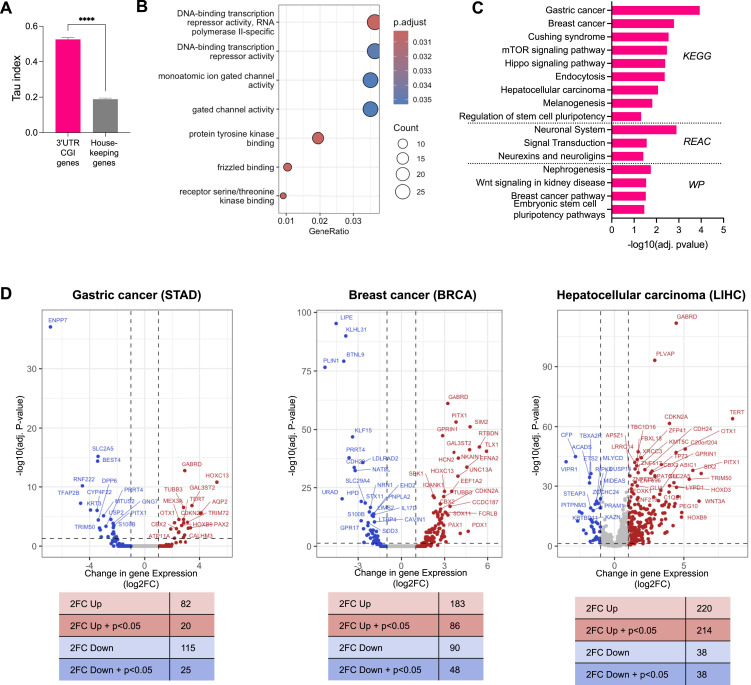
Characterisation of genes with 3′UTR CGIs. **(A)** Bar plot showing the tau index (tissue specificity; Palmer et al, 2021) of genes with 3′UTR CGIs compared with a list of housekeeping genes. Values represent the mean ± SD. ****P < 0.0001 (*t* test). **(B)** Dot plot showing gene ontology enrichment terms for genes with 3′UTR CGIs. The diameter indicates the number of genes overlapping each gene ontology term, and the colour represents the enrichment *P*-value*.* **(C)** Bar plot showing enriched pathways in the KEGG (Kanehisa et al, 2017), REAC (Milacic et al, 2024), and WP (Agrawal et al, 2024) databases for genes with 3′UTR CGIs. **(D)** Volcano plots showing differential expression of 3′UTR CGI genes in gastric cancer (STAD), breast cancer (BRCA), and hepatocellular carcinoma (LIHC) tumour tissue compared with the matched normal tissue using expression data from TCGA database. Red dots indicate genes with > twofold up-regulation, and blue dots indicate genes with > twofold down-regulation. REAC, Reactome; WP, WikiPathways; Log2FC, log_2 _fold change.

Promoter CGIs are frequently associated with ubiquitously expressed housekeeping genes, whereas only a small proportion of promoter CGIs are found at genes with tissue-specific expression. The frequent co-occurrence of 3′UTR CGIs with promoter CGIs suggests that these 3′UTR elements may provide an additional layer of regulatory control potentially influencing transcript stability, alternative polyA, or spatial and temporal expression. Their presence may allow otherwise ubiquitously expressed genes to acquire tissue-specific expression patterns. To verify this, we calculated the tissue specificity using the tau index, as described by Palmer et al (2021), and found that genes containing 3′UTR CGIs exhibited significantly higher tissue specificity scores than housekeeping genes (Fig 2A). This suggests the genes with 3′UTR CGIs may have tissue-specific functions.

To further investigate this, we performed gene ontology analysis to determine whether genes containing 3′ UTR CGIs are enriched in specific functional pathways. The analysis revealed that genes with 3′UTR CGIs are enriched for molecular functions related to transcriptional regulation and TF activity (Fig 2B), consistent with their tissue-specific expression patterns, as is often a characteristic of TFs. Interestingly, pathway analysis showed that cancer-related terms are enriched in genes with 3′UTR CGIs (Fig 2C). These terms included gastric, breast, and hepatocellular carcinoma, as well as the mTOR and Hippo signalling pathways, both of which are key pathways commonly dysregulated in cancer (Calses et al, 2019; Zou et al, 2020). Other biological processes that are known to be disrupted in tumour initiation and progression, including endocytosis (Banushi et al, 2023), melanogenesis (Slominski et al, 2022), and neuronal signalling (Keough & Monje, 2022), were also enriched.

**Figure S6.**
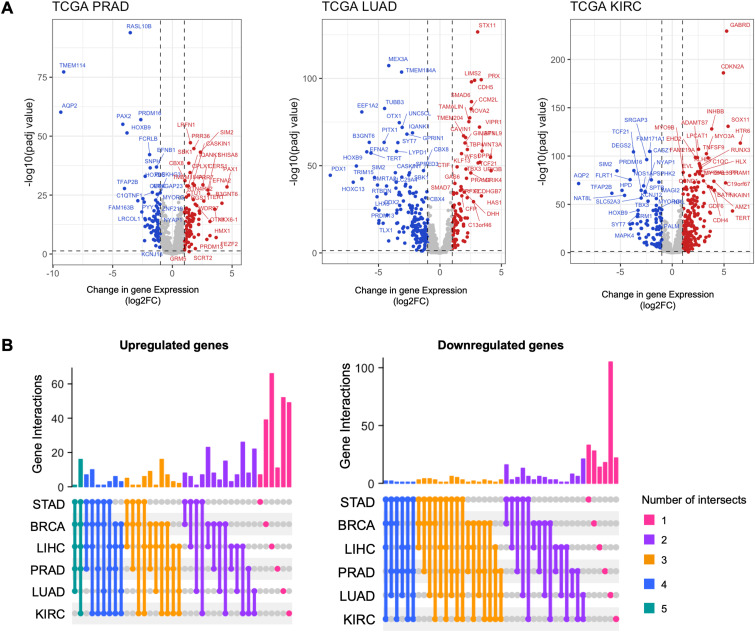
Differential expression of genes with 3′UTR CGIs in different cancer types. **(A)** Volcano plots showing differential expression of genes with 3′UTR CGIs in prostate cancer (PRAD), lung cancer (LUAD), and kidney cancer (KIRC) tumour tissues compared with normal tissues using expression data from TCGA database. Red dots indicate genes with > twofold up-regulation, and blue dots indicate genes with > twofold down-regulation. **(B)** UpSet diagrams showing the common genes with 3′UTR CGIs that are up-regulated or down-regulated across the six cancer types analysed.

Building on the functional annotation analyses, we next assessed whether genes with 3′UTR CGIs show altered expression in cancer. Given that gastric, breast, and hepatocellular carcinomas were highlighted in the pathway analysis (Fig 2C), we examined gene expression data from tumour samples from these cancer types (available via The Cancer Genome Atlas [TCGA]) and compared them with matched tissue samples. We found that a number of genes containing 3′UTR CGIs are differentially expressed across cancers but in a cancer-specific manner (Fig 2D). We then extended our expression analysis to include prostate, lung, and kidney cancers using TCGA data, and similarly observed differential expression of genes with 3′UTR CGIs (Fig S6A). However, these gene expression changes were largely cancer-specific, with relatively little overlap in up- or down-regulated 3′UTR CGI genes across the cancer types (Fig S6B).

### CGIs within 3′UTRs show altered methylation patterns in cancer

Cancer cells typically exhibit global DNA hypomethylation together with locus-specific hypermethylation of CGIs at tumour suppressor gene promoters, driving cancer-specific gene expression programmes (Nishiyama & Nakanishi, 2021). We hypothesised that like promoter CGIs, 3′UTR CGIs might also be differentially methylated in cancer. The observation that genes with 3′UTR CGIs were both differentially expressed in cancer and enriched in key cancer pathways (Fig 2) may reflect underlying alterations in 3′UTR CGI methylation.

**Figure 3.**
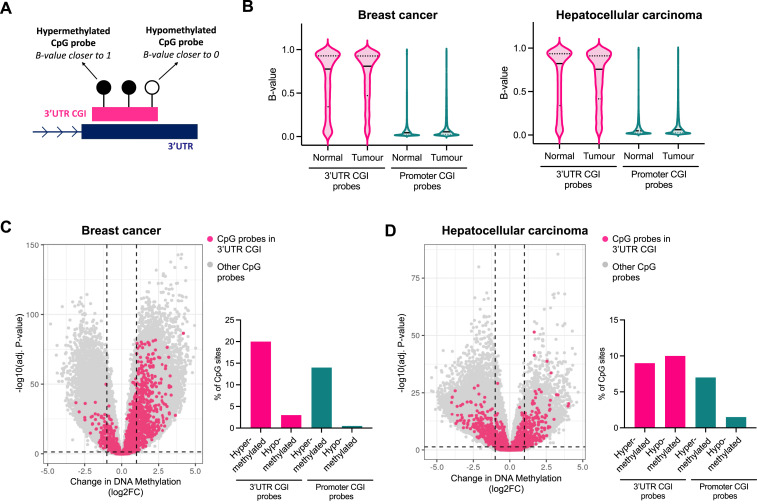
Differential methylation of 3′UTR CGIs in cancer. **(A)** Schematic illustrating how CpG probes are assessed in methylation array data. **(B)** Violin plots showing average methylation bval for CpG probes in 3′UTR CGIs and promoter CGIs in normal and tumour samples from breast cancer (normal, n = 97; tumour, n = 793) and hepatocellular carcinoma (normal, n = 50; tumour, n = 377)*.* **(C, D)** Volcano plots showing differential methylation of CpG probes in (C) breast cancer and (D) hepatocellular carcinoma tumour tissues compared with matched normal tissues. The x-axis shows log_2_FC in DNA methylation. Grey dots indicate all probes, and pink dots indicate CpG probes located in 3′UTR CGIs. Bar plots show the percentage of CpG probes that are hypermethylated or hypomethylated (> twofold change in DNA methylation) in breast cancer and hepatocellular carcinoma tumour samples. Log_2_FC, log_2_ fold change.

**Figure S7.**
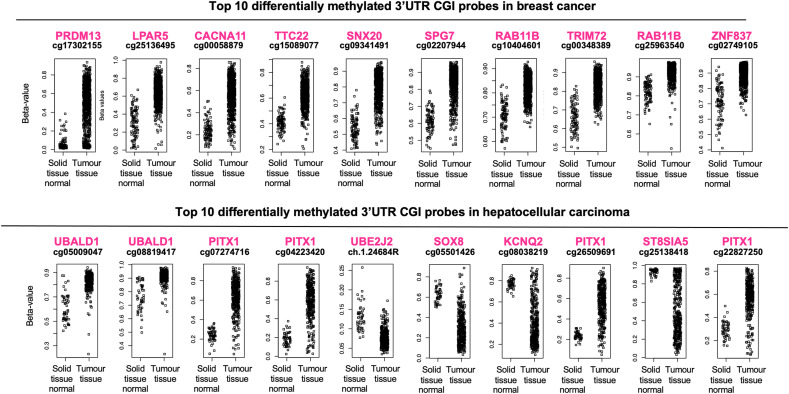
Top 10 differentially methylated 3′UTR CGI probes in breast cancer and hepatocellular carcinoma. Scatter plots showing methylation bval of the top 10 differentially methylated CpG probes located within 3′UTR CGIs in breast cancer and hepatocellular carcinoma.

Using Illumina 450K methylation array data from TCGA, we assessed β-values (bval) of individual CpG sites within both 3′UTR and promoter CGIs (Fig 3A). Across normal and tumour samples, individual CpGs in 3′UTR CGIs exhibited consistently high methylation (average β > 0.85), whereas most of the CpGs in promoter CGIs remained unmethylated in both conditions (Fig 3B). No clear difference in overall methylation levels was observed between normal and tumour samples for either CGI type, suggesting that changes may occur only at subsets of CpG sites in a cancer type–specific manner. To investigate this, we analysed differential methylation in breast cancer (BRCA) and liver cancer (LIHC) datasets. Both cancer types exhibited distinct patterns of 3′UTR CGI methylation changes (Fig 3C and D). In BRCA, CpGs in 3′UTR CGIs were more likely to be hypermethylated (∼20% hypermethylated, ∼5% hypomethylated), whereas in LIHC, both hypermethylation and hypomethylation occurred at similar levels (∼10%). To explore the potential functional impact, we identified the top 10 significantly differentially methylated CpG sites within 3′UTR CGIs and their associated genes (Fig S7). Notably, many of these genes have previously been implicated in BRCA, LIHC, or other cancers (Table S2). Collectively, these findings suggest that dynamic methylation changes at 3′UTR CGIs may contribute to cancer-associated gene regulation, although further experimental validation is required to establish causality.

Table S2. Role of genes associated with top 10 differentially methylated 3′UTR CGI probes in breast cancer and hepatocellular carcinoma.

### Differentially methylated regions within 3′UTR CGIs are linked to gene expression regulation

**Figure 4.**
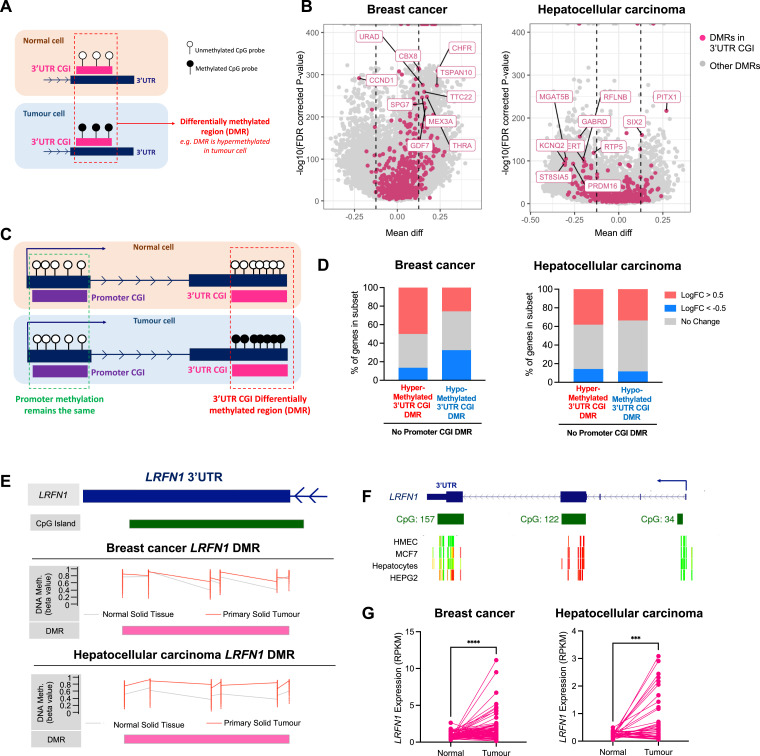
Analysis of DMRs in 3′UTR CGIs in cancers. **(A)** Schematic illustrating how DMRs are defined. **(B)** Volcano plots showing DMRs (tumour versus normal tissue) in breast cancer (normal, n= 97; tumour, n= 793) and hepatocellular carcinoma (normal, n= 50; tumour, n= 377). Genes corresponding to the top 10 DMRs are labelled (meandiff >0.125 and ranked based on *P*-value). Pink dots indicate DMRs in the 3′UTR CGI. **(C)** Schematic showing an example of a gene with a DMR in the 3′UTR CGI but no DMR in the promoter CGI region. **(D)** Bar plots showing the percentage of genes with a 3′UTR DMR but no promoter CGI DMR that are up-regulated (red, logFC > 0.5) or down-regulated (blue, logFC < −0.5) in breast cancer and hepatocellular carcinoma. **(E)** Plot showing the DMR in the 3′UTR of the *LRFN1* gene in breast cancer and hepatocellular carcinoma. Methylation levels at individual CpGs within the DMR in the tumour tissue (pink) and the corresponding normal tissue (grey) are shown. The location of the 3′UTR CGI in *LRFN1* is indicated at the top. **(F)** UCSC track showing methylated regions across *LRFN1* in HMEC (primary mammary cells), MCF7 (breast cancer cells), hepatocytes (control hepatocyte cells), and HEPG2 (hepatocellular carcinoma cells). Red lines indicate methylated regions; green lines indicate unmethylated regions. The direction of transcription and the TSS of *LRFN1* is shown by arrows on the schematic of the gene at the top. **(G)** Plot showing the expression of *LRFN1* in paired patient data from breast cancer (n = 77) and hepatocellular carcinoma dataset (n = 40). ****P < 0.0001 (paired *t* test).

Differentially methylated regions (DMRs), which consist of clusters of CpGs showing coordinated methylation changes, are known to be associated with transcriptional regulation (Campagna et al, 2021). We therefore focused on DMRs located within 3′UTR CGIs (Fig 4A). In both BRCA and LIHC, we could identify such DMRs in 3′UTRs (Fig 4B). A closer look at the genes harbouring these 3’ UTR DMRs shows that they have previously been implicated in BRCA, LIHC, or other cancers (Table S3). The pattern of hyper- and hypomethylation observed in these DMRs mirrors our results from individual CpG probes (Fig 3), with more 3′UTR CGIs hypermethylated in the BRCA tumour tissue, whereas in LIHC, both hyper- and hypomethylation were observed.

Table S3. Role of genes associated with top 10 3′UTR CGI DMRs in breast cancer and hepatocellular carcinoma.

**Figure S8.**
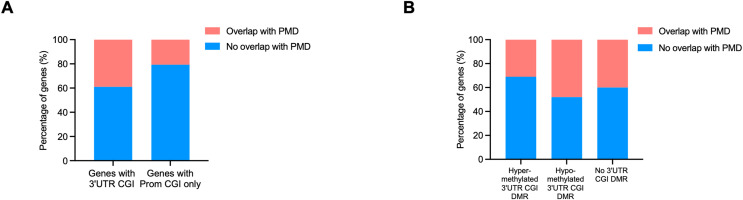
Presence of 3′UTR CGIs in PMDs. **(A)** Bar plot showing the overlap of the percentage of genes with 3′UTR CGIs and genes with only promoter CGIs that overlap PMDs in breast cancer (Brinkman et al, 2019). The red portion indicates the percentage of genes that overlap PMDs, and the blue portion indicates the percentage of genes that do not overlap PMDs. **(B)** Bar plot showing the percentage of DMRs in 3′UTR CGIs that overlap PMDs in breast cancer. 3′UTR CGI DMRs are classified as hypermethylated (> twofold increase in DNA methylation) or hypomethylated (> twofold decrease in DNA methylation).

Partially methylated domains (PMDs) are large genomic regions characterised by extensive DNA hypomethylation and are a common feature of many cancers, including BRCA (Brinkman et al, 2019). To investigate whether 3′UTR CGI–associated methylation changes occur within these broader epigenetic landscapes, we examined their overlap (Fig S8) with published PMD maps in BRCA (Brinkman et al, 2019). Genes with 3′UTR CGIs showed markedly higher overlap with PMDs (39%) compared to genes with promoter-only CGIs (21%) (Fig S8A). Further analysis (Fig S8B) revealed that genes with hypomethylated 3′UTR DMRs overlapped PMDs most frequently (48%), compared with hypermethylated DMRs (31%) or genes without DMRs (40%).

As changes in promoter methylation are known to influence gene expression profiles, we assessed gene expression changes in genes that contained a 3′UTR DMR, but did not exhibit differential methylation in their promoter CGIs (as shown in Fig 4C). This way, we could isolate the effect of methylation at 3′UTR from that of promoter methylation. In BRCA, we observed a trend whereby genes with a hypermethylated DMR in 3′ UTR showed increased expression in tumour tissue, whereas genes with a hypomethylated DMR tended to exhibit decreased expression (Fig 4D). In LIHC tumours, hypermethylated 3′UTR DMRs were similarly associated with increased gene expression, but hypomethylated DMRs did not associate with decreased expression (Fig 4D).

**Figure S9.**
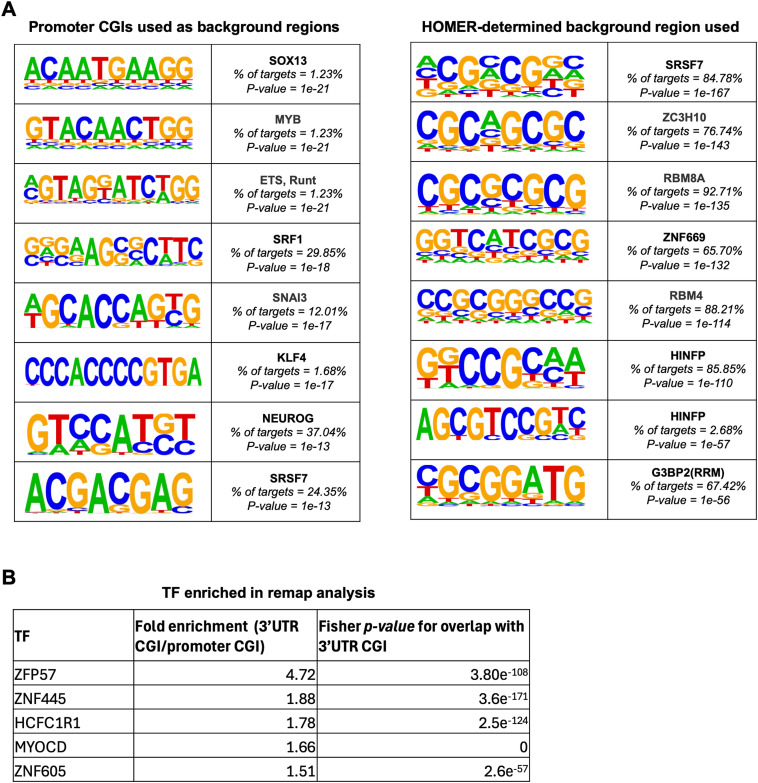
Motif and TF-binding analyses for 3′UTR CGIs. **(A)** De novo motif analysis of 3′UTR CGIs using HOMER (Heinz et al, 2010) with promoter CGIs or HOMER-determined background regions used. Motif, protein name, percentage (%) of targets, and *P*-values are shown. **(B)** Table showing TFs with >1.5-fold enrichment of binding at 3′UTR CGIs compared with promoter CGIs. The table includes fold enrichment values and *P*-values from Fisher’s exact test indicating the significance of overlap with 3′UTR CGIs.

To explore potential mechanisms by which 3′UTR CGI methylation might influence expression, we examined TF motif enrichment within 3′UTR CGIs (Fig S9A). However, motif analyses alone were insufficient to draw strong conclusions. We therefore extended this analysis using ReMap (Hammal et al, 2022), a comprehensive ChIP-seq atlas of TF binding (Fig S9B). Among the top enriched DNA-binding proteins in 3′UTR CGIs was ZFP57, showing > fourfold enrichment compared with promoter CGIs and negligible enrichment in non-CGI 3′UTRs. Given ZFP57’s role in maintaining DNA methylation (Quenneville et al, 2011), its enrichment at 3′UTR CGIs is particularly intriguing and warrants further investigation.

A clear example linking 3′UTR CGI hypermethylation with gene expression is that of the *LRFN1* gene, recently reported to have oncogenic roles in several cancers (Nikas et al, 2020; Ruan et al, 2020; Sorokin et al, 2020; Wang et al, 2022a). This gene shows a hypermethylated DMR within its 3′UTR CGI region in breast and hepatocellular carcinoma, whereas its promoter CGIs remain unchanged in methylation (Fig 4E). Consistent hypermethylation in the *LRFN1* 3′UTR is also observed in breast cancer (MCF7) and hepatocellular carcinoma (HEPG2) cell lines compared with their respective control cell lines, human mammary epithelial cells, and hepatocytes (Fig 4F). Analysis of patient data further confirms that *LRFN1* expression is elevated in BRCA and LIHC tumour tissues relative to matched normal controls (Fig 4G).

**Figure S10.**
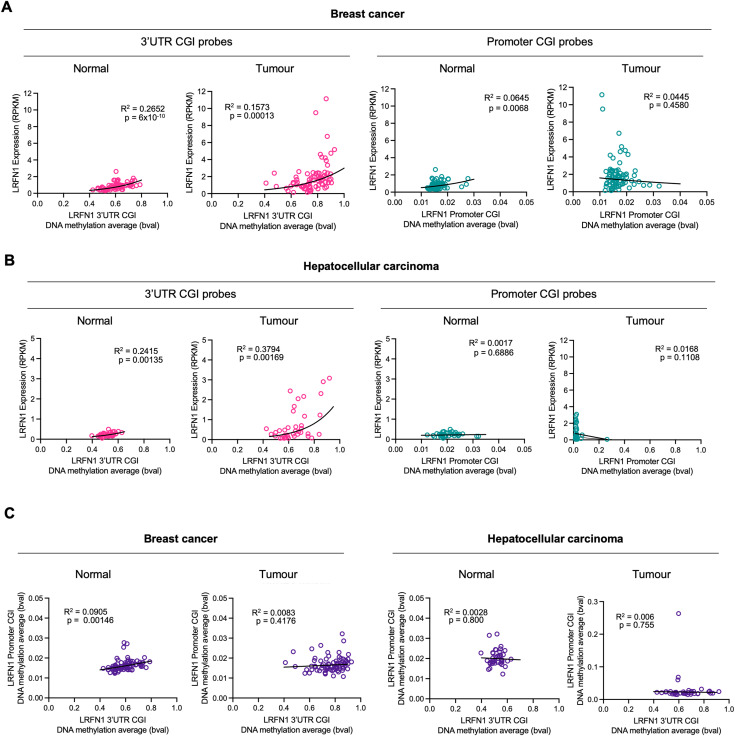
Correlation between 3′UTR and promoter CGI methylation and gene expression for the *LRFN1* gene. **(A*,* B)** Scatter plots showing the correlation between DNA methylation (based on average bval) of CpG probes within the CGIs in 3′UTRs (pink) and promoters (green) with *LRFN1* RNA expression (RPKM) in paired patient data from (A) breast cancer (n = 77) and (B) hepatocellular carcinoma dataset (n = 40). **(C)** Scatter plots showing the correlation between 3′UTR CGI and promoter CGI DNA methylation (based on average bval) within the CGIs in normal and tumour samples in breast cancer and hepatocellular carcinoma. An exponential line of best fit is plotted for all graphs with R^2^-values shown. *P*-values calculated for Pearson’s correlation between X-values and ln(Y-values), that is, to correspond to the exponential fit, are also shown.

In addition, to further explore the association between 3′UTR CGI methylation and gene expression, we performed correlation analyses for *LRFN1* as a selected example. For LRFN1, we observed a significant positive correlation between gene expression and DNA methylation at the 3′UTR CGI in both breast cancer (Fig S10A) and hepatocellular carcinoma (Fig S10B), whereas no such correlation was observed at the promoter CGI. Increased methylation at the 3′UTR CGI corresponded to an exponential increase in *LRFN1* expression, a pattern that is especially evident in tumour cells. Furthermore, no significant correlation was observed between promoter CGI methylation and 3′UTR CGI methylation in either normal or tumour samples (Fig S10C). Together, these findings provide a specific example in which 3′UTR CGI methylation is associated with increased gene expression in cancer, independently of promoter CGI methylation.

## Discussion

Most studies on CGIs have focused on their enrichment at promoter regions, where high CpG density and their DNA methylation status are linked to transcriptional regulation. Although a large proportion of CGIs are located at promoters, our analysis reveals that ∼3% of CGIs overlap 3′UTRs, at the opposite end of genes. Nearly 800 genes harbour CGIs in their 3′UTRs. Prior studies examining genome-wide CGI distribution have reported the presence of CGIs within 3′UTRs (Medvedeva et al, 2010; Chen et al, 2021; Sommerer et al, 2022). Although their work primarily examined CGI distribution, association with different genic regions, and transcriptional activity inferred from CAGE tags, they did not investigate functional pathways and DNA methylation patterns associated with these CGIs. This leaves open the important question of how site-specific DNA methylation at 3′UTR CGIs contributes to gene expression regulation.

Our analyses of 3′UTR CGIs suggest they are distinct compared with the promoter CGIs. Their unique sequence composition, enrichment in tissue-specific genes, and strong association with cancer-related signalling pathways differentiate them from classical promoter-associated CGIs. Traditionally, 3′UTRs are recognised for their role in post-transcriptional regulation; however, the extent to which 3′UTR CGIs might also influence gene expression through other mechanisms remains poorly understood. One possible way 3′UTR CGIs might function is by serving as TSSs. Previous studies have reported that more than 50% of CGIs contain known TSSs (Vavouri & Lehner, 2012) and that RNAPII shows affinity for CGIs (Deaton & Bird, 2011). However, our data provided limited support for this hypothesis, as we observed little evidence of RNAPII or the associated histone modification mark, H3K4me3, enrichment at these sites. This is consistent with a previous study on alternative promoters in liver cancer, which found that transcription typically initiates from CpG-poor regions rather than intragenic CGIs (Nepal & Andersen, 2023). Furthermore, although H3K36me3 is enriched at 3′UTR CGIs, the levels are comparable to other 3′UTRs.

Methylation of promoter CGIs is a well-established mechanism of gene regulation (Lakshminarasimhan & Liang, 2016). It is therefore plausible that 3′UTR CGIs may also be subject to methylation that influences gene regulation. Our analysis revealed that 3′UTR CGIs exhibit higher methylation levels than promoter CGIs, which are typically protected from methylation. This is particularly intriguing given that methylated CpGs are prone to mutagenesis through spontaneous deamination. The genome has lost CpGs over time because of the mutagenic nature of methylated cytosines (Poulos et al, 2017). CGIs, on the other hand, are generally thought to resist methylation, thereby maintaining their high CpG density and conservation across evolution. On this background, our observation that 3′UTR CGIs are more likely to be methylated is paradoxical. Although their smaller size and lower CpG content may be a signature of chronic methylation, their evolutionary conservation points to potential significance. This raises important questions regarding the mechanisms ensuring their preservation and the functional impact of their methylation on gene expression.

In our study, 3′UTR CGIs displayed differential methylation in tumour tissues compared with normal tissues. The overall methylation pattern of 3′UTR CGIs was cancer-specific, consistent with distinct oncogenic mechanisms. Similar observations have been reported in previous pan-cancer studies, which showed that DNA methylation signatures differ between tumour types (Yang et al, 2017; Shi et al, 2020; Ibrahim et al, 2022). In line with our results, breast cancer is globally characterised by hypomethylation as reflected in PMDs, whereas it simultaneously exhibits focal hypermethylation of CGIs. Hepatocellular carcinoma, on the other hand, shows evidence of hypomethylation (Yang et al, 2017; Ibrahim et al, 2022). Therefore, the differential 3′UTR CGI methylation patterns we observed in these cancers are in keeping with broader tumour-specific methylation landscapes.

Furthermore, in our analyses, methylated 3′UTR CGIs were associated with increased gene expression, whereas hypomethylated 3′UTR CGIs correlated with reduced expression in tumour tissues, particularly in breast cancer. The correlation between increased methylation at the 3′UTR CGI and increased transcription can be a consequence rather than the cause (Wang et al, 2022b). As a representative case study, a closer look at selected genes, such as *LRFN1*, where both tumour and control samples exhibited similar levels of gene body methylation, the 3′UTR CGI was the only region showing increased methylation in cancer. Consistent with this, *LRFN1* expression was elevated in both breast and hepatocellular carcinoma, and 3′UTR CGI methylation showed a strong positive correlation with expression, whereas promoter methylation did not exhibit such a relationship. Moreover, our observation that 3′UTR CGIs are found in tissue-specific genes rather than ubiquitously expressed housekeeping genes indicates that 3′UTR CGI methylation might provide an additional layer of specificity to gene expression. A previously published example of immune checkpoint gene *HAVCR2* supports this hypothesis (McGuire et al, 2019). Although the global relationship between 3′UTR CGI methylation and gene expression remains an open question, *LRFN1* serves as an illustrative example of the regulatory potential of these elements in a cancer context.

Interestingly, separate from the hypermethylation observed at some 3′UTR CGIs, we found that genes with hypomethylated 3′ UTR CGIs were more likely to overlap with PMDs, large genomic regions in breast cancer associated with cancer-specific hypomethylation. This suggests that although some 3′ UTR methylation changes occur as part of broader epigenomic alterations during tumorigenesis, others, such as those in *LRFN1*, appear to act in a more gene-specific manner.

Based on previous studies, we can speculate 3′UTR CGI methylation could affect transcript synthesis, processing, and stability.

One possible mechanism, suggested by a previous study, is that methylation within the 3′ UTR can promote the use of proximal polyA sites through CTCF-mediated chromatin looping (Nanavaty et al, 2020). Because the selection of alternative polyA sites determines 3′UTR length, it can, in turn, affect post-transcriptional regulation, including mRNA stability, translation efficiency, and subcellular localisation (Mayr, 2019). Another potential mechanism is through regulation of RNAPII elongation speed. Changes in RNAPII dynamics can affect splicing and polyA outcomes (Maunakea et al, 2013). Intragenic methylation has previously been shown to slow RNAPII progression, allowing for the recognition of weaker splice sites (Maunakea et al, 2013). A similar mechanism may be at play at 3′UTR CGI sites. Methylation at these loci could reduce RNAPII speed, thereby altering the choice of polyA site and favouring the production of mRNAs with shorter 3′UTRs, which are often associated with increased stability and translation.

Alternatively, methylation of the 3′UTR may influence its function by altering protein binding. Many DNA-binding factors bind to GC-rich motifs, with CGIs serving to enhance protein interactions (Deaton & Bird, 2011). Methylation of CGIs, however, can alter these interactions. Thus, it is plausible that 3′UTR CGIs act as binding sites for DNA-binding proteins or other regulators of splicing and stability. Our computational analysis provides preliminary evidence for this, revealing a marked enrichment of several TFs at 3′UTR CGIs, including ZNF445, HCFC1R1, and, most notably, ZFP57. The enrichment of TF ZFP57 at 3′UTR CGIs is particularly intriguing given its established role in maintaining DNA methylation at imprinting control regions (Takahashi et al, 2015). ZFP57 recognises methylated DNA motifs and recruits corepressors to preserve repressive epigenetic states during development (Quenneville et al, 2011; Strogantsev et al, 2015). Although it is often debated whether DNA methylation is a cause or consequence of protein binding, as seen with factors like SP1 (Lienert et al, 2011), the recruitment of ZFP57 offers a compelling case for methylation-led regulation. ZFP57 specifically recognises a methylated hexanucleotide motif and cannot associate with unmethylated DNA (Quenneville et al, 2011; Strogantsev et al, 2015). Thus, in the context of 3′UTR CGIs, the presence of DNA methylation might act as the primary signal for ZFP57 recruitment, which subsequently anchors the KAP1 corepressor complex to modulate the local epigenetic environment.

In the end, it must be acknowledged that 3′UTR CGIs present a non-canonical genomic profile. Unlike promoter CGIs, which typically exhibit a bimodal distribution of CpG density, our analysis shows that 3′UTR CGIs are part of a unimodal landscape. This suggests that statistically, these regions represent the upper tail of a continuous distribution of 3′UTR CpG content rather than a discrete structural population like in case of promoters. However, our data provide a preliminary indication that crossing this density threshold correlates with a binary shift in regulatory occupancy, as seen with the specific recruitment of ZFP57. Whether these regions represent a truly distinct class of CGI or are simply high-density 3′UTR variants remains an open question. A limitation of this study is the reliance on computational mapping and bulk sequencing data, which cannot definitively establish the causal sequence of methylation and protein recruitment. Therefore, systematic mapping and functional characterisation of other histone modifications and TFs at these regions are necessary to ascertain their direct link to gene regulation in cancer. Such work will be essential to determine whether 3′UTR CGIs function as a unique regulatory entity or primarily mirror the epigenetic signatures of their host gene bodies.

In summary, this study characterises 3′UTR CGIs as conserved features associated with tissue-specific genes and cancer-related pathways. However, the link between their conservation, unimodal CpG distribution, DNA methylation status, and enrichment of TFs such as ZFP57 remains to be further explored.

## Materials and Methods

### Identification of the 3′UTR CGI

The CGI track (Gardiner-Garden & Frommer, 1987) corresponding to human genome version hg38 was downloaded from UCSC Genome Browser (https://genome.ucsc.edu/). In this dataset, CGIs are defined as regions with a GC content ≥50%, a length >200 bp, and an observed-to-expected CpG ratio >0.6, calculated based on the number of Gs and Cs in the segment. For our analysis, we used CGI coordinates that are defined using the repeat masked version of the genome. This excludes CGIs located within repeat regions. The unmasked CGI tracks used for comparison with masked CGIs (as shown in Fig S2) were also obtained from UCSC Genome Browser.

To define the genomic locations of CGIs with respect to the gene annotations, we used the annotatePeak function from the ChIPseeker package in R (Yu et al, 2015). The promoter region was defined as −3,000 to +3,000 bp. The genomic annotations were assigned using priority order of promoter, 3′UTR, 5′UTR, exon, intron, downstream, and intergenic. Consequently, if a CGI overlapped two annotations, it was assigned to the annotation that had higher priority. For example, if a CGI overlapped both a promoter and a 5′UTR, it was assigned to the promoter because of its higher priority. This also ensured that any region annotated as a 3′UTR did not overlap a known promoter region.

### Matched control set

A matched control set of random genomic regions was generated using an in-house Python script. For each 3′UTR-associated CGI, one control region of identical length was selected using a peak-to-peak matching strategy. For each candidate interval, the proportions overlapping 3′UTR, exonic, intronic, and intergenic annotations were calculated. Candidate regions were required to closely match the corresponding 3′UTR-associated CGI with respect to these annotation fractions (default tolerance ±0.5% for each feature), and the highest-scoring candidate with the smallest overall deviation in genomic annotation composition was selected. This produced a matched control dataset of identical size and highly comparable genomic context to the original 3′UTR-associated CGI set, as illustrated in Fig S2D.

To identify genes containing 3′UTR CGIs, we used the bedtools intersect function to determine which genes from the GENCODE v43 annotation overlapped with 3′UTR CGIs (Quinlan & Hall, 2010; Frankish et al, 2019). GENCODE v43 gene annotations were used for all further analyses. To verify that 3′UTR CGI characteristics are not related to general properties of UTRs, we compared them with CGIs overlapping 5′UTRs. To perform a specific, size-matched comparison between 3′ and 5′ UTRs (Fig S3), we conducted a separate, secondary classification. In this instance, the priority was shifted to 5′UTR > Promoter > 3′UTR to capture all CGIs with any 5′UTR overlap, regardless of their proximity to a TSS. This allowed us to specifically isolate the influence of the UTR context on CGI properties.

### Characterisation of the 3′UTR CGI

To assess sequence conservation, we used the PhyloP100way scores from the UCSC Genome Browser, which provide basewise conservation estimates across 100 vertebrate species (Pollard et al, 2010). To visualise conservation patterns at 3′UTR CGIs, promoter CGIs, and the matched control set, heatmaps and metagene plots were generated using deepTools (Ramirez et al, 2016). Heatmaps were plotted centred on CGI coordinates.

The UpSet plot to identify genes containing promoter CGIs and those with 3′UTR CGIs was plotted using the *UpSet* function (Lex et al, 2014) from the Bioconductor package *ComplexHeatmap* (Gu, 2022) in R. The CGI track from the UCSC Genome Browser was used to obtain information on the CGI length, the percentage of CpG and GC content, and the observed-to-expected CpG ratio. The data corresponding to the CGI length, the percentage of CpG and GC content, and the observed-to-expected CpG ratio were plotted using GraphPad Prism. Normalised CpG densities across 3′UTRs and promoter regions were calculated as the observed-to-expected ratio (OE = [number of CpGs/(number of Cs × number of Gs)] × length of the region in nucleotides).

GENCODE v43 annotations were used to identify the nearest TSS and to plot the distance distribution relative to each 3′UTR-associated CGI. Only high-confidence transcript coordinates were considered, defined by transcript support level 1 and the presence of MANE Select or CCDS annotations.

Processed ChIP-seq data were obtained from the ENCODE database, including RNAPII (HEPG2 [GEO:GSM935603], MCF7 [GEO:GSM1006865], HeLa [GEO:GSM822273] [Zhang et al, 2020]; H3K4me3 HEPG2 [GEO:GSE96248] [ENCODE Project Consortium, 2012], H3K4me3 MCF7 [GEO:GSM945269] [Zhang et al, 2020]; H3K4me3 HeLa [GEO:GSE96127] [ENCODE Project Consortium, 2012], H3K36me3 HEPG2 [GEO:GSM733685] [Zhang et al, 2020]; H3K36me3 MCF7 [GEO:GSE174945] [ENCODE Project Consortium, 2012], H3K36me3 HeLa [GEO:GSM945230] [Thurman et al, 2012]). Metagene plots using ChIP-seq data were generated with *deepTools* (Ramirez et al, 2016).

### Characterisation of 3′UTR CGI genes

Tissue specificity of genes containing 3′UTR CGIs and housekeeping genes was assessed using the Tau Index v8 (Palmer et al, 2021). Gene ontology analysis was performed using the Bioconductor package *clusterProfiler* (Yu et al, 2012) in R. Pathway enrichment analysis was conducted using *g:Profiler* (Raudvere et al, 2019), identifying significantly enriched pathways in the KEGG (Kanehisa et al, 2017), Reactome (Milacic et al, 2024), and WikiPathways (Agrawal et al, 2024) databases.

### Analysis of expression and methylation data from TCGA

Gene expression and clinical data for gastric cancer (STAD), breast cancer (BRCA), hepatocellular carcinoma (LIHC), prostate cancer (PRAD), lung cancer (LUAD), and kidney cancer (KIRC) were downloaded from TCGA (Tomczak et al, 2015) using the Bioconductor package TCGAbiolinks (Colaprico et al, 2016) in R. Differential gene expression analysis was performed using DESeq2 (Love et al, 2014) in R.

For DNA methylation analysis, BRCA and LIHC 450K methylation array data and associated clinical data were downloaded using *TCGAbiolinks*. Differential analysis of CpG probes was performed using the Bioconductor package *limma* (Ritchie et al, 2015) in R. For each CpG probe, a linear model was fitted to the intensity values across all samples using the lmFit function, and statistics and *P*-values for differential methylation were computed using the eBayes function. The top 10 differentially methylated probes located within 3′UTR CGIs were visualised using the plotCpg function. DMRs were identified and analysed using the Bioconductor package *DMRcate* (Peters et al, 2015) with a bandwidth of 1,000 nucleotides (lambda = 1,000) and a scaling factor of 2 (C = 2), as recommended for array data. DMRs were plotted using the Bioconductor package *Gviz* (Hahne & Ivanek, 2016) in R. CpG methylation data from 450K Bead Arrays for cell lines HMEC, MCF7, hepatocytes, and HepG2 (ENCODE/HAIB) were visualised using the UCSC Genome Browser.

For the *LRFN1* gene, expression (RPKM) was compared between paired normal and tumour samples from BRCA and LIHC datasets. DNA methylation values for the *LRFN1* 3′UTR and promoter CGIs were calculated using bval from 450K methylation array data. As multiple CpG probes map to each CGI, the average bval across the 3′UTR and promoter CGIs was computed for each sample. Correlation analyses were then performed to assess the relationship between average CGI methylation (bval) and *LRFN1* expression (RPKM) in normal and tumour samples for both cancer types. Graphs were generated using GraphPad Prism, and exponential lines of best fit were plotted. The corresponding R^2^-values and *P*-values calculated using Pearson’s correlation on linearised values using ln(y) were reported.

### Motif analysis

To identify TF motifs within 3′UTR CGIs, the *findMotifsGenome.pl* function in HOMER (Heinz et al, 2010) was used with the -noweight and -size options. Promoter CGIs and HOMER-defined background regions were used for comparison. The resulting output included the motif, associated protein name, percentage of target sequences containing the motif, and corresponding *P-*value. As an orthogonal approach, we also used ReMap (Hammal et al, 2022), a database of experimentally determined TF-binding regions, to assess TF enrichment at 3′UTR CGIs.

For each TF in the ReMap dataset, Fisher’s exact test was performed to evaluate whether TF-binding sites are significantly enriched in 3′UTR CGIs compared with promoter CGIs. The analysis was done using the bedtools fisher function, which counts overlaps between TF-binding site coordinates and CGI coordinates.

To quantify the magnitude of enrichment, a fold enrichment metric was calculated as follows:

$$\text{Fold}\;\text{enrichment}=\frac{\text{Fraction}\;\text{of}\;3’\text{UTR}\;\text{CGIs}\;\text{overlapping}\;\text{TF binding}\;\text{sites}}{\text{Fraction}\;\text{of}\;\text{promoter}\;\text{CGIs}\;\text{overlapping}\;\text{TF binding}\;\text{sites}}.$$

This fold enrichment indicates whether a TF preferentially binds 3′UTR CGIs relative to promoter CGIs.

### Statistical analysis

Statistical analyses were performed using GraphPad Prism or the built-in functions of the computational tools used. To assess significance, Brown–Forsythe’s and Welch’s ANOVA tests were used (Figs 1E and S2B), whereas paired *t* tests were applied where appropriate (Figs 2A, 4G, and S3A). *P*-values are reported in the figure legends or indicated on the figures, with values <0.05 considered statistically significant.

## Supplementary Material

Reviewer comments

## Data Availability

The following publicly available datasets were analysed during the current study. CGI track (Gardiner-Garden & Frommer, 1987) was downloaded from UCSC Genome Browser (https://genome.ucsc.edu) (hg38). Processed ChIP-seq data were accessed from the ENCODE database (https://www.encodeproject.org). RNAPII HEPG2 [GEO:GSM935603], RNAPII MCF7 [GEO:GSM1006865], RNAPII HeLa [GEO:GSM822273], H3K4me3 MCF7 [GEO:GSM945269], and H3K36me3 HEPG2 [GEO:GSM733685] can be accessed via Zhang et al (2020). H3K4me3 HEPG2 [GEO:GSE96248], H3K4me3 HeLa [GEO:GSE96127], and H3K36me3 MCF7 [GEO:GSE174945] can be accessed via ENCODE Project Consortium (2012). H3K36me3 HeLa [GEO:GSM945230] can be accessed via Thurman et al (2012). RNA-seq, 450K methylation array data, and clinical data for gastric cancer (STAD), breast cancer (BRCA), hepatocellular carcinoma (LIHC), prostate cancer (PRAD), lung cancer (LUAD), and kidney cancer (KIRC) were downloaded from TCGA (Tomczak et al, 2015).

## References

[bib1] Agrawal A, Balci H, Hanspers K, Coort SL, Martens M, Slenter DN, Ehrhart F, Digles D, Waagmeester A, Wassink I, (2024) Wikipathways 2024: Next generation pathway database. Nucleic Acids Res 52: D679–D689. 10.1093/nar/gkad960 37941138 PMC10767877

[bib2] Akhtar MM, Scala G, Cocozza S, Miele G, Monticelli A (2013) CpG islands under selective pressure are enriched with H3K4me3, H3K27ac and H3K36me3 histone modifications. BMC Evol Biol 13: 145. 10.1186/1471-2148-13-145 23837650 PMC3711888

[bib3] Amante SM, Montibus B, Cowley M, Barkas N, Setiadi J, Saadeh H, Giemza J, Contreras-Castillo S, Fleischanderl K, Schulz R, (2020) Transcription of intragenic CpG islands influences spatiotemporal host gene pre-mRNA processing. Nucleic Acids Res 48: 8349–8359. 10.1093/nar/gkaa556 32621610 PMC7470969

[bib4] Banushi B, Joseph SR, Lum B, Lee JJ, Simpson F (2023) Endocytosis in cancer and cancer therapy. Nat Rev Cancer 23: 450–473. 10.1038/s41568-023-00574-6 37217781

[bib5] Bird AP (1986) CpG-rich islands and the function of DNA methylation. Nature 321: 209–213. 10.1038/321209a0 2423876

[bib6] Brinkman AB, Nik-Zainal S, Simmer F, Rodriguez-Gonzalez FG, Smid M, Alexandrov LB, Butler A, Martin S, Davies H, Glodzik D, (2019) Partially methylated domains are hypervariable in breast cancer and fuel widespread CpG island hypermethylation. Nat Commun 10: 1749. 10.1038/s41467-019-09828-0 30988298 PMC6465362

[bib7] Cain JA, Montibus B, Oakey RJ (2022) Intragenic CpG islands and their impact on gene regulation. Front Cell Dev Biol 10: 832348. 10.3389/fcell.2022.832348 35223855 PMC8873577

[bib8] Calses PC, Crawford JJ, Lill JR, Dey A (2019) Hippo pathway in cancer: Aberrant regulation and therapeutic opportunities. Trends Cancer 5: 297–307. 10.1016/j.trecan.2019.04.001 31174842

[bib9] Campagna MP, Xavier A, Lechner-Scott J, Maltby V, Scott RJ, Butzkueven H, Jokubaitis VG, Lea RA (2021) Epigenome-wide association studies: Current knowledge, strategies and recommendations. Clin Epigenetics 13: 214. 10.1186/s13148-021-01200-8 34863305 PMC8645110

[bib10] Chan JJ, Tabatabaeian H, Tay Y (2023) 3’UTR heterogeneity and cancer progression. Trends Cell Biol 33: 568–582. 10.1016/j.tcb.2022.10.001 36372614

[bib11] Chen Y, Xia F, Jiang B, Wang W, Li X (2021) Role of immune cell-specific hypermethylation signatures in classification and risk stratification of breast cancer. Front Med (Lausanne) 8: 674338. 10.3389/fmed.2021.674338 34513864 PMC8426625

[bib12] Colaprico A, Silva TC, Olsen C, Garofano L, Cava C, Garolini D, Sabedot TS, Malta TM, Pagnotta SM, Castiglioni I, (2016) Tcgabiolinks: An R/bioconductor package for integrative analysis of TCGA data. Nucleic Acids Res 44: e71. 10.1093/nar/gkv1507 26704973 PMC4856967

[bib13] ENCODE Project Consortium (2012) An integrated encyclopedia of DNA elements in the human genome. Nature 489: 57–74. 10.1038/nature11247 22955616 PMC3439153

[bib14] Deaton AM, Bird A (2011) Cpg islands and the regulation of transcription. Genes Dev 25: 1010–1022. 10.1101/gad.2037511 21576262 PMC3093116

[bib15] Derti A, Garrett-Engele P, Macisaac KD, Stevens RC, Sriram S, Chen R, Rohl CA, Johnson JM, Babak T (2012) A quantitative atlas of polyadenylation in five mammals. Genome Res 22: 1173–1183. 10.1101/gr.132563.111 22454233 PMC3371698

[bib16] Frankish A, Diekhans M, Ferreira AM, Johnson R, Jungreis I, Loveland J, Mudge JM, Sisu C, Wright J, Armstrong J, (2019) GENCODE reference annotation for the human and mouse genomes. Nucleic Acids Res 47: D766–D773. 10.1093/nar/gky955 30357393 PMC6323946

[bib17] Gardiner-Garden M, Frommer M (1987) CpG islands in vertebrate genomes. J Mol Biol 196: 261–282. 10.1016/0022-2836(87)90689-9 3656447

[bib18] Gu Z (2022) Complex heatmap visualization. Imeta 1: e43. 10.1002/imt2.43 38868715 PMC10989952

[bib19] Hahn MA, Wu X, Li AX, Hahn T, Pfeifer GP (2011) Relationship between gene body DNA methylation and intragenic h3k9me3 and h3k36me3 chromatin marks. PLoS One 6: e18844. 10.1371/journal.pone.0018844 21526191 PMC3079728

[bib20] Hahne F, Ivanek R (2016) Visualizing genomic data using Gviz and bioconductor. Methods Mol Biol 1418: 335–351. 10.1007/978-1-4939-3578-9_16 27008022

[bib21] Hammal F, de Langen P, Bergon A, Lopez F, Ballester B (2022) Remap 2022: A database of human, mouse, *Drosophila* and *Arabidopsis* regulatory regions from an integrative analysis of DNA-binding sequencing experiments. Nucleic Acids Res 50: D316–D325. 10.1093/nar/gkab996 34751401 PMC8728178

[bib22] Heinz S, Benner C, Spann N, Bertolino E, Lin YC, Laslo P, Cheng JX, Murre C, Singh H, Glass CK (2010) Simple combinations of lineage-determining transcription factors prime cis-regulatory elements required for macrophage and b cell identities. Mol Cell 38: 576–589. 10.1016/j.molcel.2010.05.004 20513432 PMC2898526

[bib23] Hong D, Jeong S (2023) 3’UTR diversity: Expanding repertoire of RNA alterations in human mRNAs. Mol Cells 46: 48–56. 10.14348/molcells.2023.0003 36697237 PMC9880603

[bib24] Hoque M, Ji Z, Zheng D, Luo W, Li W, You B, Park JY, Yehia G, Tian B (2013) Analysis of alternative cleavage and polyadenylation by 3’ region extraction and deep sequencing. Nat Methods 10: 133–139. 10.1038/nmeth.2288 23241633 PMC3560312

[bib25] Hughes AL, Kelley JR, Klose RJ (2020) Understanding the interplay between cpg island-associated gene promoters and h3k4 methylation. Biochim Biophys Acta Gene Regul Mech 1863: 194567. 10.1016/j.bbagrm.2020.194567 32360393 PMC7294231

[bib26] Ibrahim J, Op de Beeck K, Fransen E, Peeters M, Van Camp G (2022) Genome-wide DNA methylation profiling and identification of potential pan-cancer and tumor-specific biomarkers. Mol Oncol 16: 2432–2447. 10.1002/1878-0261.13176 34978357 PMC9208075

[bib27] Jeziorska DM, Murray RJS, De Gobbi M, Gaentzsch R, Garrick D, Ayyub H, Chen T, Li E, Telenius J, Lynch M, (2017) DNA methylation of intragenic CpG islands depends on their transcriptional activity during differentiation and disease. Proc Natl Acad Sci U S A 114: E7526–E7535. 10.1073/pnas.1703087114 28827334 PMC5594649

[bib28] Kanehisa M, Furumichi M, Tanabe M, Sato Y, Morishima K (2017) Kegg: New perspectives on genomes, pathways, diseases and drugs. Nucleic Acids Res 45: D353–D361. 10.1093/nar/gkw1092 27899662 PMC5210567

[bib29] Keough MB, Monje M (2022) Neural signaling in cancer. Annu Rev Neurosci 45: 199–221. 10.1146/annurev-neuro-111020-092702 35259916 PMC10234771

[bib30] Lakshminarasimhan R, Liang G (2016) The role of DNA methylation in cancer. Adv Exp Med Biol 945: 151–172. 10.1007/978-3-319-43624-1_7 27826838 PMC7409375

[bib31] Lex A, Gehlenborg N, Strobelt H, Vuillemot R, Pfister H (2014) Upset: Visualization of intersecting sets. IEEE Trans Vis Comput Graph 20: 1983–1992. 10.1109/TVCG.2014.2346248 26356912 PMC4720993

[bib32] Lienert F, Wirbelauer C, Som I, Dean A, Mohn F, Schubeler D (2011) Identification of genetic elements that autonomously determine DNA methylation states. Nat Genet 43: 1091–1097. 10.1038/ng.946 21964573

[bib33] Long HK, King HW, Patient RK, Odom DT, Klose RJ (2016) Protection of CpG islands from DNA methylation is DNA-encoded and evolutionarily conserved. Nucleic Acids Res 44: 6693–6706. 10.1093/nar/gkw258 27084945 PMC5001583

[bib34] Love MI, Huber W, Anders S (2014) Moderated estimation of fold change and dispersion for RNA-seq data with DESeq2. Genome Biol 15: 550. 10.1186/s13059-014-0550-8 25516281 PMC4302049

[bib35] Maunakea AK, Chepelev I, Cui K, Zhao K (2013) Intragenic DNA methylation modulates alternative splicing by recruiting MeCP_2_ to promote exon recognition. Cell Res 23: 1256–1269. 10.1038/cr.2013.110 23938295 PMC3817542

[bib36] Mayr C (2019) What are 3’ UTRs doing? Cold Spring Harb Perspect Biol 11: a034728. 10.1101/cshperspect.a034728 30181377 PMC6771366

[bib37] Mayya VK, Duchaine TF (2019) Ciphers and executioners: How 3’-untranslated regions determine the fate of messenger RNAs. Front Genet 10: 6. 10.3389/fgene.2019.00006 30740123 PMC6357968

[bib38] McGuire MH, Herbrich SM, Dasari SK, Wu SY, Wang Y, Rupaimoole R, Lopez-Berestein G, Baggerly KA, Sood AK (2019) Pan-cancer genomic analysis links 3’UTR DNA methylation with increased gene expression in T cells. EBioMedicine 43: 127–137. 10.1016/j.ebiom.2019.04.045 31056473 PMC6558231

[bib39] Medvedeva YA, Fridman MV, Oparina NJ, Malko DB, Ermakova EO, Kulakovskiy IV, Heinzel A, Makeev VJ (2010) Intergenic, gene terminal, and intragenic CpG islands in the human genome. BMC Genomics 11: 48. 10.1186/1471-2164-11-48 20085634 PMC2817693

[bib40] Milacic M, Beavers D, Conley P, Gong C, Gillespie M, Griss J, Haw R, Jassal B, Matthews L, May B, (2024) The reactome pathway knowledgebase 2024. Nucleic Acids Res 52: D672–D678. 10.1093/nar/gkad1025 37941124 PMC10767911

[bib41] Moore LD, Le T, Fan G (2013) DNA methylation and its basic function. Neuropsychopharmacology 38: 23–38. 10.1038/npp.2012.112 22781841 PMC3521964

[bib42] Nanavaty V, Abrash EW, Hong C, Park S, Fink EE, Li Z, Sweet TJ, Bhasin JM, Singuri S, Lee BH, (2020) DNA methylation regulates alternative polyadenylation via CTCF and the cohesin complex. Mol Cell 78: 752–764.e6. 10.1016/j.molcel.2020.03.024 32333838 PMC7245569

[bib43] Nepal C, Andersen JB (2023) Alternative promoters in CpG depleted regions are prevalently associated with epigenetic misregulation of liver cancer transcriptomes. Nat Commun 14: 2712. 10.1038/s41467-023-38272-4 37169774 PMC10175279

[bib44] Nikas JB, Mitanis NT, Nikas EG (2020) Whole exome and transcriptome RNA-sequencing model for the diagnosis of prostate cancer. ACS Omega 5: 481–486. 10.1021/acsomega.9b02995 31956794 PMC6964263

[bib45] Nishiyama A, Nakanishi M (2021) Navigating the DNA methylation landscape of cancer. Trends Genet 37: 1012–1027. 10.1016/j.tig.2021.05.002 34120771

[bib46] Palmer D, Fabris F, Doherty A, Freitas AA, de Magalhaes JP (2021) Ageing transcriptome meta-analysis reveals similarities and differences between key mammalian tissues. Aging (Albany NY) 13: 3313–3341. 10.18632/aging.202648 33611312 PMC7906136

[bib47] Peters TJ, Buckley MJ, Statham AL, Pidsley R, Samaras K, Lord RV, Clark SJ, Molloy PL (2015) De novo identification of differentially methylated regions in the human genome. Epigenetics Chromatin 8: 6. 10.1186/1756-8935-8-6 25972926 PMC4429355

[bib48] Pollard KS, Hubisz MJ, Rosenbloom KR, Siepel A (2010) Detection of nonneutral substitution rates on mammalian phylogenies. Genome Res 20: 110–121. 10.1101/gr.097857.109 19858363 PMC2798823

[bib49] Poulos RC, Olivier J, Wong JWH (2017) The interaction between cytosine methylation and processes of DNA replication and repair shape the mutational landscape of cancer genomes. Nucleic Acids Res 45: 7786–7795. 10.1093/nar/gkx463 28531315 PMC5737810

[bib50] Quenneville S, Verde G, Corsinotti A, Kapopoulou A, Jakobsson J, Offner S, Baglivo I, Pedone PV, Grimaldi G, Riccio A, (2011) In embryonic stem cells, ZFP_57_/KAP_1_ recognize a methylated hexanucleotide to affect chromatin and DNA methylation of imprinting control regions. Mol Cell 44: 361–372. 10.1016/j.molcel.2011.08.032 22055183 PMC3210328

[bib51] Quinlan AR, Hall IM (2010) Bedtools: A flexible suite of utilities for comparing genomic features. Bioinformatics 26: 841–842. 10.1093/bioinformatics/btq033 20110278 PMC2832824

[bib52] Ramirez F, Ryan DP, Gruning B, Bhardwaj V, Kilpert F, Richter AS, Heyne S, Dundar F, Manke T (2016) deepTools2: A next generation web server for deep-sequencing data analysis. Nucleic Acids Res 44: W160–W165. 10.1093/nar/gkw257 27079975 PMC4987876

[bib53] Raudvere U, Kolberg L, Kuzmin I, Arak T, Adler P, Peterson H, Vilo J (2019) g:Profiler: A web server for functional enrichment analysis and conversions of gene lists (2019 update). Nucleic Acids Res 47: W191–W198. 10.1093/nar/gkz369 31066453 PMC6602461

[bib54] Ritchie ME, Phipson B, Wu D, Hu Y, Law CW, Shi W, Smyth GK (2015) Limma powers differential expression analyses for RNA-sequencing and microarray studies. Nucleic Acids Res 43: e47. 10.1093/nar/gkv007 25605792 PMC4402510

[bib55] Ruan B, Feng X, Chen X, Dong Z, Wang Q, Xu K, Tian J, Liu J, Chen Z, Shi W, (2020) Identification of a set of genes improving survival prediction in kidney renal clear cell carcinoma through integrative reanalysis of transcriptomic data. Dis Markers 2020: 8824717. 10.1155/2020/8824717 33110456 PMC7578724

[bib56] Saxonov S, Berg P, Brutlag DL (2006) A genome-wide analysis of CpG dinucleotides in the human genome distinguishes two distinct classes of promoters. Proc Natl Acad Sci U S A 103: 1412–1417. 10.1073/pnas.0510310103 16432200 PMC1345710

[bib57] Shi M, Tsui SK, Wu H, Wei Y (2020) Pan-cancer analysis of differential DNA methylation patterns. BMC Med Genomics 13: 154. 10.1186/s12920-020-00780-3 33087120 PMC7579968

[bib58] Siepel A, Bejerano G, Pedersen JS, Hinrichs AS, Hou M, Rosenbloom K, Clawson H, Spieth J, Hillier LW, Richards S, (2005) Evolutionarily conserved elements in vertebrate, insect, worm, and yeast genomes. Genome Res 15: 1034–1050. 10.1101/gr.3715005 16024819 PMC1182216

[bib59] Slominski RM, Sarna T, Plonka PM, Raman C, Brozyna AA, Slominski AT (2022) Melanoma, melanin, and melanogenesis: The Yin and Yang relationship. Front Oncol 12: 842496. 10.3389/fonc.2022.842496 35359389 PMC8963986

[bib60] Sommerer Y, Ohlei O, Dobricic V, Oakley DH, Wesse T, Sedghpour Sabet S, Demuth I, Franke A, Hyman BT, Lill CM, (2022) A correlation map of genome-wide DNA methylation patterns between paired human brain and buccal samples. Clin Epigenetics 14: 139. 10.1186/s13148-022-01357-w 36320053 PMC9628033

[bib61] Sorokin M, Poddubskaya E, Baranova M, Glusker A, Kogoniya L, Markarova E, Allina D, Suntsova M, Tkachev V, Garazha A, (2020) RNA sequencing profiles and diagnostic signatures linked with response to ramucirumab in gastric cancer. Cold Spring Harb Mol Case Stud 6: a004945. 10.1101/mcs.a004945 32060041 PMC7133748

[bib62] Steinhaus R, Gonzalez T, Seelow D, Robinson PN (2020) Pervasive and CpG-dependent promoter-like characteristics of transcribed enhancers. Nucleic Acids Res 48: 5306–5317. 10.1093/nar/gkaa223 32338759 PMC7261191

[bib63] Strogantsev R, Krueger F, Yamazawa K, Shi H, Gould P, Goldman-Roberts M, McEwen K, Sun B, Pedersen R, Ferguson-Smith AC (2015) Allele-specific binding of ZFP_57_ in the epigenetic regulation of imprinted and non-imprinted monoallelic expression. Genome Biol 16: 112. 10.1186/s13059-015-0672-7 26025256 PMC4491874

[bib64] Takahashi N, Gray D, Strogantsev R, Noon A, Delahaye C, Skarnes WC, Tate PH, Ferguson-Smith AC (2015) ZFP_57_ and the targeted maintenance of postfertilization genomic imprints. Cold Spring Harb Symp Quant Biol 80: 177–187. 10.1101/sqb.2015.80.027466 27325708

[bib65] Thurman RE, Rynes E, Humbert R, Vierstra J, Maurano MT, Haugen E, Sheffield NC, Stergachis AB, Wang H, Vernot B, (2012) The accessible chromatin landscape of the human genome. Nature 489: 75–82. 10.1038/nature11232 22955617 PMC3721348

[bib66] Tomczak K, Czerwinska P, Wiznerowicz M (2015) The Cancer Genome Atlas (TCGA): An immeasurable source of knowledge. Contemp Oncol (Pozn) 19: A68–A77. 10.5114/wo.2014.47136 25691825 PMC4322527

[bib67] Vavouri T, Lehner B (2012) Human genes with CpG island promoters have a distinct transcription-associated chromatin organization. Genome Biol 13: R110. 10.1186/gb-2012-13-11-r110 23186133 PMC3580500

[bib68] Wang H, Liu J, Yang J, Wang Z, Zhang Z, Peng J, Wang Y, Hong L (2022a) A novel tumor mutational burden-based risk model predicts prognosis and correlates with immune infiltration in ovarian cancer. Front Immunol 13: 943389. 10.3389/fimmu.2022.943389 36003381 PMC9393426

[bib69] Wang Q, Xiong F, Wu G, Liu W, Chen J, Wang B, Chen Y (2022b) Gene body methylation in cancer: Molecular mechanisms and clinical applications. Clin Epigenetics 14: 154. 10.1186/s13148-022-01382-9 36443876 PMC9706891

[bib70] Yang X, Gao L, Zhang S (2017) Comparative pan-cancer DNA methylation analysis reveals cancer common and specific patterns. Brief Bioinform 18: 761–773. 10.1093/bib/bbw063 27436122

[bib71] Yu G, Wang LG, Han Y, He QY (2012) Clusterprofiler: An R package for comparing biological themes among gene clusters. OMICS 16: 284–287. 10.1089/omi.2011.0118 22455463 PMC3339379

[bib72] Yu G, Wang LG, He QY (2015) ChIPseeker: An R/bioconductor package for ChIP peak annotation, comparison and visualization. Bioinformatics 31: 2382–2383. 10.1093/bioinformatics/btv145 25765347

[bib73] Zhang J, Lee D, Dhiman V, Jiang P, Xu J, McGillivray P, Yang H, Liu J, Meyerson W, Clarke D, (2020) An integrative ENCODE resource for cancer genomics. Nat Commun 11: 3696. 10.1038/s41467-020-14743-w 32728046 PMC7391744

[bib74] Zou Z, Tao T, Li H, Zhu X (2020) MTOR signaling pathway and mTOR inhibitors in cancer: Progress and challenges. Cell Biosci 10: 31. 10.1186/s13578-020-00396-1 32175074 PMC7063815

