# Supplementary material for: Investigating the role of CpG islands and DNA methylation at 3′UTRs in cancer
Source: Life Sci Alliance. 2026 Jul 24;9(10):e202503234. doi: 10.26508/lsa.202503234 (PMC13401056; doi:10.26508/lsa.202503234)
Supplement: Reviewer comments [file LSA-2025-03234_review_history.pdf]

Life Science Alliance

Investigating the role of CpG islands and DNA methylation at 3'UTRs in cancer

Claire Wilson and Aditi Kanhere
DOI: https://doi.org/10.26508/lsa.202503234

Corresponding author(s): Dr. Aditi Kanhere (University of Liverpool)

Review Timeline:

Submission Date:	2025-01-23
Editorial Decision:	2025-03-26
Revision Received:	2025-11-04
Editorial Decision:	2025-12-12
Revision Received:	2025-05-27
Editorial Decision:	2026-06-23
Accepted:	2026-07-15

Scientific Editor: Eric Sawey, PhD and Tim Fessenden, PhD

Transaction Report:

(Note: With the exception of the correction of typographical or spelling errors that could be a source of ambiguity, letters and reports are not edited. The original formatting of letters and referee reports may not be reflected in this compilation.)

No full Peer Review Process File is available with this article, as the authors have chosen not to make the review process public in this case.

March 26, 2025

Re: Life Science Alliance manuscript #LSA-2025-03234-T

Aditi Kanhere
University of Liverpool, Institute of Systems, Molecular & Integrative Biology

Dear Dr. Kanhere,

Thank you for submitting your manuscript entitled "Investigating the role of CpG island DNA methylation at 3'UTRs in cancer" to Life Science Alliance. The manuscript was assessed by expert reviewers, whose comments are appended to this letter. We invite you to submit a revised manuscript addressing the Reviewer comments.

To upload the revised version of your manuscript, please log in to your account: https://lsa.msubmit.net/cgi-bin/main.plex

You will be guided to complete the submission of your revised manuscript and to fill in all necessary information. Please get in touch in case you do not know or remember your login name.

While you are revising your manuscript, please also attend to the below editorial points to help expedite the publication of your manuscript. Please direct any editorial questions to the journal office.

The typical timeframe for revisions is three months. Please note that papers are generally considered through only one revision cycle, so strong support from the referees on the revised version is needed for acceptance.

When submitting the revision, please include a letter addressing the reviewers' comments point by point.

We hope that the comments below will prove constructive as your work progresses.

Thank you for this interesting contribution to Life Science Alliance. We are looking forward to receiving your revised manuscript.

Sincerely,

Eric Sawey, PhD
Executive Editor
Life Science Alliance
http://www.lsajournal.org

A. THESE ITEMS ARE REQUIRED FOR REVISIONS

-- A letter addressing the reviewers' comments point by point.

-- An editable version of the final text (.DOC or .DOCX) is needed for copyediting (no PDFs).

-- High-resolution figure, supplementary figure and video files uploaded as individual files: See our detailed guidelines for preparing your production-ready images, https://www.life-science-alliance.org/authors

-- Summary blurb (enter in submission system): A short text summarizing in a single sentence the study (max. 200 characters including spaces). This text is used in conjunction with the titles of papers, hence should be informative and complementary to the title and running title. It should describe the context and significance of the findings for a general readership; it should be written in the present tense and refer to the work in the third person. Author names should not be mentioned.

-- By submitting a revision, you attest that you are aware of our payment policies found here: https://www.life-science-alliance.org/copyright-license-fee

B. MANUSCRIPT ORGANIZATION AND FORMATTING:

Full guidelines are available on our Instructions for Authors page, https://www.life-science-alliance.org/authors

We encourage our authors to provide original source data, particularly uncropped/-processed electrophoretic blots and spreadsheets for the main figures of the manuscript. If you would like to add source data, we would welcome one PDF/Excel-file per figure for this information. These files will be linked online as supplementary "Source Data" files.

IMPORTANT: It is Life Science Alliance policy that if requested, original data images must be made available. Failure to provide original images upon request will result in unavoidable delays in publication. Please ensure that you have access to all original microscopy and blot data images before submitting your revision.

December 12, 2025

Re: Life Science Alliance manuscript #LSA-2025-03234-TR

Dr. Aditi Kanhere
University of Liverpool
Molecular and Clinical Cancer Medicine
Institute of Systems, Integrative and Molecular Biology, University of Liverpool
Liverpool, United Kingdom L69 3GE
United Kingdom

Dear Dr. Kanhere,

Thank you for submitting your revised manuscript entitled "Investigating the role of CpG island and DNA methylation at 3'UTRs in cancer" to Life Science Alliance. We appreciate your patience while we considered a decision, which was significantly slowed by editor availability. The manuscript has been seen by Reviewers 1 and 3 whose comments are appended below, whereas Reviewer 2 was unavailable and was previously overall supportive. As you will see, Reviewers 1 and 3 both express overlapping and significant concerns. Unfortunately Reviewer 3 expresses lower confidence in this revised manuscript than in the earlier version.

These reviewers remain unconvinced that the CGI analysis here unveils a bone fide epigenetic pattern that is truly specific to 3' UTR regions. This concern was exacerbated by the new Fig S1, highlighted by Reviewer 3, whose results are consistent with methylation events that overlap the 3' end of the coding region. This proximity in addition to the short length of these regions complicate the analysis, ultimately leaving these reviewers unconvinced by the central conclusions reached including on evolutionary conservation.

However, we feel that the data presented in this work remain valuable to readers if properly contextualized and interpreted more carefully. We are open to considering a revised manuscript that acknowledges limitations inherent to this analysis and presents findings as preliminary in the absence of causal evidence for the proposed roles of 3' UTR methylation patterns. A revised manuscript must confront the concerns especially of Reviewer 3 throughout the text (abstract, results, discussion). This must include an

expanded discussion of the observation in Fig S1 to clearly convey that CGIs examined here have overlap with coding regions. The conclusions on sequence conservation must be similarly tempered, again per Reviewer 3. If you wish to include further analysis to allay the concerns of these reviewers you are welcome to include those, but journal policy is for a single major round of major revisions.

LSA strives to offer fast consideration as a service to authors, and I regret that is not possible in this case. If you wish to pursue publication of this work in its current form, it would be best to seek a different outlet.

Should you wish to pursue consideration at LSA by revising the text, please submit the final revision within one month, along with a letter that includes a point by point response to the remaining reviewer comments. I strongly suggest we discuss the reviewer concerns and the requested revisions via email or videoconferencing. Please contact me to set this up.

To upload the revised version of your manuscript, please log in to your account:
https://lsa.msubmit.net/cgi-bin/main.plex
You will be guided to complete the submission of your revised manuscript and to fill in all necessary information.

Please pay attention to adhere to our editorial requirements for revisions:

A. THESE ITEMS ARE REQUIRED FOR REVISIONS

-- A letter addressing the reviewers' comments point by point.

-- An editable version of the final text (.DOC or .DOCX) is needed for copyediting (no PDFs).

-- High-resolution figure, supplementary figure and video files uploaded as individual files: See our detailed guidelines for preparing your production-ready images, https://www.life-science-alliance.org/authors

-- Summary blurb (enter in submission system): A short text summarizing in a single sentence the study (max. 200 characters including spaces). This text is used in conjunction with the titles of papers, hence should be informative and complementary to the title and running title. It should describe the context and significance of the findings for a general readership; it should be written in the present tense and refer to the work in the third person. Author names should not be mentioned.

B. MANUSCRIPT ORGANIZATION AND FORMATTING:

Full guidelines are available on our Instructions for Authors page, https://www.life-science-alliance.org/authors

Thank you for this interesting contribution to Life Science Alliance.

Sincerely,

Tim Fessenden
Scientific Editor
Life Science Alliance

June 23, 2026

RE: Life Science Alliance Manuscript #LSA-2025-03234-TRR

Dr. Aditi Kanhere
University of Liverpool
Molecular and Clinical Cancer Medicine
Institute of Systems, Integrative and Molecular Biology, University of Liverpool
Liverpool, United Kingdom L69 3GE
United Kingdom

Dear Dr. Kanhere,

Thank you for submitting your revised manuscript entitled "Investigating the role of CpG island and DNA methylation at 3'UTRs in cancer". Our previous decision invited a second revision, on the basis of concerns of Reviewers 1 and 3 weighed against the support of Reviewer 2.

Unfortunately Reviewer 3 was unavailable to assess this revision. This reviewer mainly questioned if authors were analyzing the entire CGI or solely the portion that overlaps the 3' UTR . Changes implemented in the revised manuscript address this concern by clarifying the focus of the analysis is on entire CGIs that overlap 3' UTRs and including additional analyses on the effects of repeat element masking.

We are grateful to the detailed assessment by Reviewer 1 and their continued engagement which has markedly improved this work. This reviewer remains unconvinced of the major claim that 3' UTR CGIs represent a distinct regulatory element, different from the rest of 3' UTR regions. This reviewer questions the relevance of analyzing these elements. They are unconvinced by the transcription factor binding analysis and conservation analysis. In addition, this reviewer pointed to observations on gene expression changes at one locus (LRFN1) which did not address their overarching concern. Overall this reviewer disputes the methods or the results from analysis they had previously requested while remaining unenthusiastic about the overall framing of this work.

The concern that the work does not separate the regulatory roles of 3' UTRs from CGI 3' UTRs is somewhat in tension with the critique from Reviewer 3 that actually the work did not appropriately consider CGIs separate from 3' UTRs. The revised manuscript

confronts this concern from Reviewer 3 and we feel this matter has been appropriately addressed. We appreciate that the current manuscript has not resolved all open questions, and that the work softens its claims on these elements as a bone fide regulatory feature of gene regulation. Our view is that readers can evaluate for themselves from the totality of evidence and interpretation if the analysis of 3' UTR CGIs, separate from 3' UTRs and from CGIs more broadly, retains relevance for human biology.

With this determination, we would be happy to publish your paper in Life Science Alliance pending adjusting the wording on methylation-expression analysis of LRFN1, addition of methods details on conservation analysis, and final revisions necessary to meet our formatting guidelines.

MANUSCRIPT ORGANIZATION AND FORMATTING:

To avoid unnecessary delays in the acceptance and publication of your paper, please read the following information carefully. Full guidelines are available on our Instructions for Authors page, https://www.life-science-alliance.org/authors

Submission of a paper that does not conform to Life Science Alliance guidelines will delay the acceptance of your manuscript.

-Please upload your Tables in editable .doc or Excel format.
-Please add callouts for Figures S8A-B; S9A-B and S10A-C to your main manuscript text.

We encourage our authors to provide original source data, particularly uncropped/-processed electrophoretic blots and spreadsheets for the main figures of the manuscript. If you would like to add source data, we would welcome one PDF/Excel-file per figure for this information. These files will be linked as supplementary "Source Data" files.

It is Life Science Alliance policy that if requested, original data images must be made available to the editors. Failure to provide original images upon request will result in unavoidable delays in publication. Please ensure that you have access to all original data images prior to final submission.

If you are planning a press release on your work, please inform us immediately to allow informing our production team and scheduling a release date.

We welcome submissions of potential cover images for the issue of LSA in which your work would appear. If you have high quality images associated with this work, please feel free to email these, with a caption, to the journal office.

LSA encourages authors to provide a 30-60 second video where the study is briefly explained. These videos will be appear embedded with the manuscript online at Life Science Alliance, and on social media to promote the published paper and authors (for examples, see https://docs.google.com/document/d/1-UWCfbE4pGcDdcgzcmiuJl2XMBJnxKYeqRvLLrLSo8s/edit?usp=sharing). Corresponding or first-authors are welcome to submit the video. Please submit only one video per manuscript. The video can be emailed to contact@life-science-alliance.org

FINAL FILES:

To upload the final version of your manuscript, please log in to your account: https://lsa.msubmit.net/cgi-bin/main.plex
You will be guided to complete the submission of your revised manuscript and to fill in all necessary information. Please get in touch in case you do not know or remember your login name.

The following items are required for acceptance.

-- An editable version of the final text (.DOC or .DOCX) is needed for copyediting (no PDFs).

-- High-resolution figure, supplementary figure and video files uploaded as individual files: See our detailed guidelines for preparing your production-ready images, https://www.life-science-alliance.org/authors

-- Summary blurb (enter in submission system): A short text summarizing in a single sentence the study (max. 200 characters including spaces). This text is used in conjunction with the titles of papers, hence should be informative and complementary to the title. It should describe the context and significance of the findings for a general readership; it should be written in the present tense and refer to the work in the third person. Author names should not be mentioned.

The license to publish form must be signed before your manuscript can be sent to production. A link to the license to publish form will be available to the corresponding author only. Please take a moment to check your funder requirements.

Reviews, decision letters, and point-by-point responses associated with peer-review at Life Science Alliance will be published, alongside the manuscript. If you do want to opt out of having the reviewer reports and your point-by-point responses displayed, please let us know immediately.

Thank you for your attention to these final processing requirements. Please revise and format the manuscript and upload materials as soon as you are able.

Thank you for this interesting contribution to the literature. We look forward to publishing your paper in Life Science Alliance.

Sincerely,

Tim Fessenden
Scientific Editor
Life Science Alliance

July 15, 2026

RE: Life Science Alliance Manuscript #LSA-2025-03234-TRRR

Dr. Aditi Kanhere
University of Liverpool
Molecular and Clinical Cancer Medicine
Institute of Systems, Integrative and Molecular Biology, University of Liverpool
Liverpool, United Kingdom L69 3GE
United Kingdom

Dear Dr. Kanhere,

Thank you for submitting your Research Article entitled "Investigating the role of CpG islands and DNA methylation at 3'UTRs in cancer". It is a pleasure to let you know that your manuscript is now accepted for publication in Life Science Alliance. Congratulations on this interesting work, which represents a long and concerted effort from your group to resolve reviewer concerns.

The final published version of your manuscript will be deposited by us to PubMed Central upon publication.

Your manuscript will now progress through copyediting and proofing. It is journal policy that authors provide original data upon request.

Your article will publish open access upon publication under a CC-BY license.

Reviews, decision letters, and point-by-point responses associated with peer-review at Life Science Alliance will be published, alongside the manuscript. If you do want to opt out of having the reviewer reports and your point-by-point responses displayed, please let us know immediately.

IMPORTANT: If you will be unreachable at any time, please provide us with the email address of an alternate author. Failure to respond to routine queries may lead to unavoidable delays in publication.

Scheduling details will be available from our production department. You will receive proofs shortly before the publication date. Only essential corrections can be made at the

proof stage so if there are any minor final changes you wish to make to the manuscript, please let the journal office know now.

DISTRIBUTION OF MATERIALS:
Authors are required to distribute freely any materials used in experiments published in Life Science Alliance. Authors are encouraged to deposit materials used in their studies to the appropriate repositories for distribution to researchers.

You can contact the journal office with any questions, contact@life-science-alliance.org

Again, congratulations on a very nice paper. I hope you found the review process to be constructive and are pleased with how the manuscript was handled editorially. We look forward to future exciting submissions from your lab.

Sincerely,

Tim Fessenden
Scientific Editor
Life Science Alliance
